# Gel-Forming Soil Conditioners of Combined Action: Field Trials in Agriculture and Urban Landscaping

**DOI:** 10.3390/polym14235131

**Published:** 2022-11-25

**Authors:** Andrey V. Smagin, Nadezhda B. Sadovnikova, Elena A. Belyaeva, Victoria N. Krivtsova, Sergey A. Shoba, Marina V. Smagina

**Affiliations:** 1 Soil Science Department and Eurasian Center for Food Security, Lomonosov Moscow State University, GSP-1, Leninskie Gory, 119991 Moscow, Russia; 2 Institute of Forest Science, Russian Academy of Sciences (ILAN), 21, Sovetskaya, Moscow Region, 143030 Uspenskoe, Russia

**Keywords:** synthetic polymer hydrogels, soil functioning regimes, water saving, antipathogenic protection, biodegradation, salinity protection, environmental monitoring, crop growing, urban landscaping, mathematical modeling

## Abstract

The article summarizes multivariate field trials of gel-forming soil conditioners for agriculture and urban landscaping in various climatic conditions from arid (O.A.E., Uzbekistan) to humid (Moscow region, Russia). The field test program included environmental monitoring of weather data, temperature, water–air regimes, salinity, alkalinity, and biological activity of various soils (sandy and loamy sandy Arenosols, Retisols, loamy Serozems), productivity and yield of plants (lawns, vegetables) and their quality, including pathogen infestation. The evolutionary line of polymer superabsorbents from radiation-crosslinked polyacrylamide (1995) to the patented “Aquapastus” material (2014–2020) with amphiphilic fillers and biocidal additives demonstrated not only success, but also the main problems of using hydrogels in soils (biodegradation, osmotic collapse, etc.), as well as their technological solutions. Along with innovative materials, our know-how consisted in the intelligent soil design of capillary barriers for water accumulation and antipathogenic and antielectrolyte protection of the rhizosphere. Gel-forming polymer conditioners and new technologies of their application increase the productivity of plant crops and the quality of biomass by 30–50%, with a 1.3–2-fold saving of water resources and reliable protection of the topsoil from pathogens and secondary salinization. The results can be useful to a wide range of specialists from chemical technologists to agronomists and landscapers.

## 1. Introduction

Gel-forming composite conditioners are actively used in modern agronomy and landscaping to optimize water retention, water conductivity, aggregation, and ion-exchange capacity of soils and their erosion resistance, as well as to fix agrochemicals and plant-protection products in the rhizosphere [1,2,3,4,5,6,7]. Hydrogel’s working doses (0.05–0.5% by weight) are ten or even a hundred times lower in comparison with traditional ameliorants used in amounts of 1–10% and more [8,9,10,11,12]. These doses effectively increase the water retention of soils by 3–5 times, increase the storage of productive water in topsoil by 1.5–2 times, and reduce water evaporation by 1.3–3 times and unproductive infiltration losses by 3–10 times [4,8,11,12,13,14]. The same and even smaller doses of gel-forming polymeric materials strongly aggregate the soil particles and protect them from wind erosion [7,12,13]. For finely dispersed soils, a small additive (10 mg/L) of water-soluble anionic polyacrylamide in irrigation water significantly affects infiltration and water absorption from irrigation furrows, structuring and protecting the soil from water erosion [6]. The use of gel-forming superabsorbents provides a 1.5–2-fold or more increase in seed germination, the prolongation of the survival time of grass and woody plants under water stress, and the growth of root and underground biomass of plants and their total yield, along with the possibility of a 1.3–2-fold saving of water resources [4,13,14]. Along with the optimization of the water regime, gel-forming composite materials can be successfully used as agents for systems of retention and the controlled release of agrochemicals and pesticides [2,3,4,5,8,15]. A sharp (up to 10 times or more) reduction in infiltration water losses, along with the retention of water-soluble fertilizers (NPK), trace elements, and pesticides in the liquid phase inside the polymer mesh, as well as ion exchange and adsorption due to chemical and physical interaction with the polymer matrix and its fillers, reliably protect the active substance from leaching [3,4,15]. Unlike the traditionally used solid-phase carriers (e.g., peat, zeolites, perlite, vermiculite), the application of gel-forming conditioners for pesticides and agrochemicals fixation in the root area is more effective because they are localized not on the surface, but in the entire volume of the carrier. Consequently, the protection of roots and tubers with gel compositions should be more advanced because of a tight and all-round (i.e., not pointy) contact of plant organs with antipathogenic biocides [4]. Technologies of nanostructural organization and architecture of composite polymer materials, as well as the synthesis of smart gels, are actively used to create various forms of composite materials (microgels, nanocapsules, films, nanosheets, multilayer gel membranes, etc.), including polymers with a changing structure for controlled release of the retained substance (nutrients, water) under the influence of temperature, *pH*, electricity, and other controlling factors [2,5,16,17,18]. The intercalation of organic and inorganic biocides in composite acrylic superabsorbents, as well as biomimetic technologies for the synthesis of polymethacrylates with a cationic and membranolytic biocidal effect, allow materials to be obtained for the environmentally friendly control of pathogens and eutrophits with low effective concentrations *E*_50_ of biocides near 10–100 ppm (soil) and 100–6000 ppb (water) [4,19].

At the same time, a number of environmental factors can negatively affect gel-forming soil conditioners and decrease their efficiency as superabsorbents of water and solutes [4,8,20,21]. First of all, there is the suppression of swelling of hydrogels in the rigid pore space of soils and under the influence of the osmotic pressure of the soil solution or irrigation water, then the syneresis of gel structures, and finally, their biodegradation under the action of soil microorganisms [4,6,8,20,21,22,23]. From chemical structural formulas of acrylic acid and polyacrylamide, hydrogels contain about 50 wt % carbon and up to 19 wt % nitrogen, which indicates a high potential biodegradability of these organic materials according to a well-known C/N criterion [24]. However, despite this obvious fact, and rare publications confirming the high biodegradability of synthetic polymer hydrogels [4,20,25,26,27,28,29], most reviews support the opinion about their absolute stability as opposed to biodegradable hydrogels based on biopolymers, mainly polysaccharides [2,5,30]. In most cases, negative environmental factors are completely ignored and only the positive effects of new composite materials are studied, mainly in laboratory experiments. Comprehensive field tests of synthesized materials in real soil and climatic conditions are carried out quite rarely [31,32,33,34,35,36,37,38,39,40,41]. Their results are often contradictory, especially regarding the effective doses of gel-forming soil conditioners [4,34,37,41]. Taking into account this situation, we have set the goal of this research to obtain and analyze the results of comprehensive field trials of composite gel-forming soil conditioners for agriculture and landscaping in different soil and climatic conditions.

We started this research in 1995 with radiation-crosslinked polyacrylamide [42], a widely used soil conditioner to improve water retention and erosion protection [43]. However, despite its success in extra-arid conditions, this polymer revealed two of the most serious problems limiting the effectiveness of acrylic gel-forming soil conditioners, namely their rapid biodegradation and osmotic suppression of swelling in saline soils and mineralized irrigation water [21,22,23]. Therefore, our further developments were aimed at solving these problems in two main directions. The first is the development of composite gel-forming soil conditioners of combined action that are more resistant to adverse environmental factors based on the filling of an acrylic polymer matrix with amphiphilic components and biocidal additives [8]. The second is the intelligent soil design of capillary barriers based on computer simulation of the “soil-gel-plant-atmosphere” system to substantiate the optimal doses, application methods, and depths of the location for gel-forming materials in the designed soil construction [42]. The result of these long-term developments was a line of gel-forming soil conditioners patented in 2014–2020 in the Russian Federation with the Aquapastus trademark, combining good technological properties (high water retention, dispersity, aggregate strength, enrichment with trace elements, and bactericidal and fungicidal properties) with resistance to biodegradation and osmotic or load suppression of swelling in the pore space of the soil [8]. The composition and technological properties of these materials, as well as the methodology of laboratory technological tests of composite gel-forming soil conditioners, were discussed in our previous publication [8]. Simultaneously, we have developed and improved the methodology of field testing of gel-forming soil conditioners using modern instrumental methods and equipment for environmental monitoring of soil regimes, productivity, and yield of plants [44].

This article summarizes all these long-term studies in order to comprehensively characterize the effectiveness of the practical use of gel-forming soil conditioners for agriculture and urban landscaping, along with limiting problems. The most important achievement of this study is the practical confirmation of the combined conditioning effect of new composite materials and technologies that not only effectively increase water retention, reduce unproductive water losses with reliable fixation of root nutrition elements and fungicides in the rhizosphere, but also protect the topsoil from pathogenic organisms and secondary salinization. The presence of amphiphilic ionogenic groups and univalent metal ions in the polymer matrix of new composite materials, along with the mechanical reinforcement of the polymer network with dispersed particles, increases the resistance of gel structures to osmotic collapse and mechanical stress, as shown in [8]. Biocidal additives, as well as the location of polymeric materials at a distance from the soil surface, increase their resistance to biodegradation and prolong their service life in the soil. Regardless of climatic conditions (arid, humid zones), tested plant crops and soils, new composite materials, and technologies for their use allow for the achievement of a 30–50% increase in plant productivity (yield), good survival of plant material, and effective antipathogenic protection at 1.3–2 times savings of irrigation water in arid irrigated agriculture, or a similar prolongation of productivity during dry periods in a humid climate, along with protection of the root layer from secondary salinization.

## 2. Materials and Methods

### 2.1. Gel-Forming Soil Conditioners

The acrylic composite materials tested in field experiments were presented by a consistently modernizing line from radiation-crosslinked technical polyacrylamide (PAA) to innovative products of the Ural Chemical Plant (Russian Federation, Perm) under the Aquapastus trademark [8]. Radiation-crosslinked PAA with the degree of swelling of 700–1000 g H_2_O per 1 g of dry material in pure water was synthesized in the Institute of Chemical Physics, Russian Academy of Sciences) [22]. This superabsorbent was prepared using the γ-radiation crosslinking of linear acrylamide and acrylic acid in a 10% water solution at a ^60^Co γ-radiation dose of 0.85 Mrad. The content of acrylic acid in the original copolymer was 10 mol %. The know-how of Aquapastus innovative products consisted in the use of a patented technology [8] for the synthesis of composite gel-forming soil conditioners with the filling of the acrylic polymer matrix by natural amphiphilic ingredients (dispersed peat, humates), as well as the introduction of microelements and antipathogenic protection agents in the form of organic and inorganic biocides. Its prototype is the basic technology of copolymerization in solution or free radical-initiated polymerization of acrylic acid salts with acrylamide and a crosslinking agent, mainly used in the production of superabsorbents [45]. We used Metylene-bis-acrylamide as a crosslinking agent and aqueous solutions of ammonium persulfate and sodium sulfate as initiators [8]. An obligatory hydrophilic component of innovative composite materials, Aquapastus is a polymer matrix based on acrylamide (AA) and salts of acrylic acid (Ak) represented by ammonium acrylate and sodium acrylate in different ratios of copolymers. This polymer base provides the formation of gel structures in pure aqueous and slightly mineralized solutions with high water absorption from 300 to 1000 g/g. Preliminary laboratory tests [8] allowed us to choose the following variants of gel-forming conditioners for manufacturing in quantities of about 100 kg, sufficient for field trials. In the base hydrophilic material Aquapastus-11 (the A11 gel), an acrylic polymer matrix with a ratio of AA/Ak from 23/75% to 40/60% was filled by 28% (mass) biocatalytic wastes from the production of polyacrylamide. Its modification includes 1% additive of silver ions in the form of nitrate (the A11-Ag gel) as inhibitor of polymer’s biodegradation and agents of antipathogenic protection. In the second innovative material, Aquapastus-22 (the A22 or “black“ gel), a similar polymer matrix was filled by 24% (mass) dispersed peat, as the most accessible and cheap Russian natural biopolymer with amphiphilic properties necessary for soil aggregation and retention of organic pesticides. This hydrogel also could presumably form more stably to pressure gel structures of the reinforced type due to the fine-dispersed filler [8,46]. Its modification (the A22-Ag gel) also contained technological additive to the polymer matrix in the form of 1% of silver ions. Additionally, we used silver ions, nanoparticles, and Quadris fungicide based on Azoxystorobin (Syngenta-group, Switzerland, Basel; https://www.syngenta.com, accessed on 20 October 2022), which were introduced in doses of 50–100 ppm not into the polymer matrix, but into the intermicellar solution during the preswelling of the gel before its application in the soil, according to [4]. The injected colloidal silver was represented by the experimental product Zeroxxe (AgroChimProm-group, Kazakhstan, Almaty; http://tdahp.ru/en/, accessed on 20 October 2022), which contains silver nanoparticles with a size of 10–70 nm superficially modified with environmentally safe biodegradable amphiphilic surfactant (tallow amphopolycarboxyglycinate-stabilizer) [47]. Prior to application to the soil, gel structures were obtained by free 24 h swelling of the dry hydrogels in pure water or in the solutions containing water-soluble or suspended biocides, with a “dry gel: liquid phase” ratio from 1:100 to 1:200, according to [4]. Detailed descriptions of the synthesis, composition, and results of preliminary laboratory testing of the Aquapastus composites for soil conditioning are presented in our previous articles [4,8] and patents (see section “Patents” at the end of the article).

### 2.2. Experimental Sites and Design

Field experiments testing gel-forming soil conditioners for sustainable agriculture and urban landscaping were carried out during the summer growing seasons in arid (U.A.E., Dubai), (Uzbekistan, Tashkent region) and humid (Russia, Moscow region) climatic conditions under natural precipitation and different types of irrigation (sprinkling, drip, flood irrigation). At all experimental sites, the complete randomized block design included several different treatments and an untreated control, each in triplicate [4]. The experimental unit with individual treatment consisted of a 2–5 m wide by 10–20 m long plot separated from adjacent plots with a 0.5–1 m wide buffer strip. The first experiment was conducted within the framework of the Russian–Arab project “Green Wave” in 1995 in the U.A.E. at the Dubai Municipality Department of Horticulture and Public Parks Station (N 25.236850, E 55.325432) with a test lawn culture *Paspalum vaginatum* (plantation density near 15–16 seedling/m^2^) under sprinkle irrigation by the use of stationary irrigated system controlled automatically by watering timers [42]. The climate of the coastal territory of the Persian Gulf countries is desert with a dry season of more than 4 months with natural precipitation of about 100 mm/year and extremely high temperatures in the daytime (from 30 °C in summer and from 15–20 °C in winter). The soil is carbonate loamy–sandy Arenosol (the dominant particle size is 100–250 µm, Table 1) with a low content of organic carbon (*C*_org_ = 0.3%), slightly higher alkalinity (*pH* = 8.1), and salinity of the liquid phase (*EC* = 5.1 dS/m), according to the Richards soil salinity guidelines [48]. The swollen (1:200) PAA hydrogel was applied at a dose of 0.1% to a 10 cm soil layer with mechanical stirring and then covered with a 5 cm layer of natural agricultural sand. This treatment ensured the formation of an imperfect capillary barrier, according to [49], increasing soil water retention and reducing unproductive infiltration losses of water. The experiments were accompanied by environmental monitoring of meteorological parameters, indicators of the dynamics of the content and storage of soil water, its electrical conductivity (relative salt concentration), irrigation water consumption and its unproductive losses, soil respiration (carbon dioxide emissions), morphometric and weight indicators of lawn grass growth (size and mass of aboveground and underground plant organs), and its quality (content chlorophyll).

Field testing of gel-forming soil conditioners for urban landscaping with growing grassy lawns continued in a humid climate under the State contract of the Moscow Government in the Serebryanyi Bor experimental station of the RAS Institute of Forest Science (Moscow Western Administrative District; 55.77090274 N, 37.39450062 E) [42]. Moscow is located in the central taiga forest region of the boreal belt, and its climatic conditions are temperate continental. The average annual amplitude of air temperature is 28 °C. Annual precipitation varies on average within 540–650 mm; the duration of the winter period is 160–170 days. The “heat island” effect, global climate change, and other anthropogenic causes lead to an increase in temperatures, especially extreme ones, reaching 30–40 °C or more in some years in summer, as well as a synchronous water deficit that can last for a month or more. These facts, along with the excessive use of antifreeze agents in the form of easily soluble salts (application rates of 1 kg/m^2^ and more), form a trend of convergence of conditions for landscaping in the Moscow metropolis and cities in the arid zone. The cultivated urbo–soddy–podzolic soil (Retisol) of the experimental station of the previous desert Arenosol (loamy sand, Table 1) has a high content of organic carbon (*C*_org_ = 3.8%), an acid reaction (*pH* = 5.2), and low electrical conductivity (*EC* = 0.5 dS/m), indicating the absence of easily soluble salts. In the experiments of 2010, we tested A11 hydrogel applied at a dose of 0.2% in the form of a 10 cm capillary barrier (similar treatment with the experiment in Dubai) under a turf grass mixture (Eurograss, *Lolium perenne* L., *Festuca rubra* L., *Poa pratensis* L.) with a seeding rate of 50 g/m^2^ and a one-time water-charging irrigation of 120 mm and artificial salinization (6 g/L NaCl solution in the same dose of 120 mm or 0.72 kg/m^2^ of leachable salt). Additionally, another capillary barrier was formed on part of the territory at a depth of 50 cm in the form of a 10 cm layer of crushed stone. To fully block secondary salinization, a perfect capillary barrier was formed in part of the territory, completely breaking the capillarity between topsoil and subsoil. At this site, the soil was removed to a depth of 50 cm using a JCB universal tractor with a qualified operator. At the bottom of the formed bed, crushed stone was placed (the maximum size of the individual stones up to 5 cm) with a layer of 10 cm. The original loamy–sandy soil (topsoil) was returned to the crushed stone layer covered with geotextile. Topsoil was also treated with A 11 hydrogel, as on the site without a crushed stone screen.

The same territory of the forest experimental station in the Moscow region was used for experiments in 2017, 2018, and 2020 with potato cultivation under the influence of gel-forming soil conditioners combining water retention and antipathogenic protection of the rhizosphere. The 2017 open-field experiment without irrigation was detailed in our previous article [4], and we did not repeat its analysis in this publication. A follow-up 2018 experiment with the Red Scarlett potato cultivar was conducted in a closed polycarbonate greenhouse with automatic ventilation and 8 automated drip irrigation lines controlled by GA-319N electronic timers (China) for irrigation management and Equatel-SVK-15G water flowmeters (Russia). The experiment included the following treatments: untreated control (without hydrogels, watering due to moisture deficit, or 100% watering time), A22 hydrogel composition with fungicide “Quadris” (50 ppm), A22 hydrogel composition with silver ions (100 ppm), and A11 hydrogel composition with silver nanoparticles (100 ppm), in doses of 0.5 and 1.0 L hydrogels per potato bush, and an their arrangement as a continuous layer without mixing and with uniform mixing of the hydrogel and the soil in equal volumes. In this experiment, biocidal compositions were prepared by introducing ions, silver nanoparticles, and organic fungicide into a solution to swell polymers immediately before they were introduced into the soil in the form of gel structures. Doses of biocides from 50 to 100 pm meant their concentration in the gel structure relative to the volume of absorbed water at a swelling degree of 1:100. The experimental plots had two drip irrigation treatments: normal (100% watering time) and economical (50% watering time relative to untreated control).

A similar experiment in a closed greenhouse with automated drip irrigation was repeated on the same sites in 2020. This experiment was designed to test new composite hydrogels, A11-Ag and A22-Ag, with silver embedded in the polymer matrix. The experimental design included untreated control and treatment with hydrogel A22 without silver and with composite hydrogels A11-Ag and A22-Ag containing embedded silver at a dose of 1%. This dose gave a concentration of 100 ppm of silver in the gel structure with a swelling degree of 1:100. All air conditioners were applied in economically optimal identical volumes of 0.5 L per bush, followed by mixing with a mineral soil substrate when planting Red Scarlett potato tubers.

Two additional experiments in 2018 and 2020 were carried out in arid climatic conditions of Uzbekistan (Tashkent region, Qumaryk farm (N 41.06613390, E 69.33949355)) with potato varieties Gala and Santana on typical Serozem (loam, Table 1; *C_org_* = 2.1%; *pH* = 7.8; *EC* = 3.8 dS/m) with traditional for Central Asia flood irrigation along the furrows. Here, on an experimental site with a total area of 1.3 hectares, we tested protective gel compositions A11, A11-Ag, and A22-Ag, similar to the Moscow experiment, in the form of swollen (1:100) gel structures in two doses of 0.5 and 1 l for every 0. 5 linear meters of furrow, with furrow irrigation at amounts of 50 and 100% applied watering rate. In 2018, the swollen gel was applied in a continuous layer without mixing with the soil; in 2020, it was applied with mixing.

Fieldwork included mechanical processing of the site (plowing, harrowing, cutting furrows), incorporation and planting of potatoes and hydrogels, periodic weeding and loosening (4–8 times per season), hilling, watering, preharvest cutting of potato tops, harvesting with a differentiated assessment of the amount, mass, and linear dimensions (length, width) of potato tubers. When growing potatoes, morphometric manual control of the height (*H*) of the bushes was carried out, as well as instrumental control of the hydrothermal parameters of the atmosphere and soil (temperature, relative humidity, precipitation), soil moisture (water–air regime index), *pH* (acid–base regime) in the upper (0–5 cm) layer and in the rhizosphere (10–20 cm), soil respiration, and pathogenic damage to the original planting material and fresh crop in accordance with the methods and criteria presented in [4,44].

### 2.3. Instrumental Methods and Equipment; Data Processing

Automated hydrothermal monitoring of temperature (*T*, [°C]) and relative humidity (*RH*, [%]) was carried out by programmable loggers “Hygrochron” DS1923 (Dallas Semiconductor, Dallas, TX, USA). The water content (*W*, [%]), electrical conductivity (*EC*, [dS/m]), and *pH* in the soil were evaluated by loggers Decagon with sensors 5TE (Decagon Devices Inc., WA, USA), the combined EU/TDS/*pH* meter HANNA HI 98129 Combo (HANNA Instruments Deutschland GmbH, Fleringen, Germany), and the combined *pH*/moisture meter AMTAST AMT-300 (Amtast USA Inc., Lakeland, FL. USA) based on the TDR method, conductometry and potentiometry. The exception was the 1995 experiment, with manual measurements of water content and electrical conductivity on samples taken by a soil auger, followed by drying in the laboratory and standard thermal-weight analysis of water content, conductometric analysis of electrical conductivity in a state of soil saturation with water, according to the Richards method [48]. To calculate the water storage and the index of the water–air regime (*W*/*W_s_*) according to [50], the bulk density of the soil (ρ*_b_*, [g/cm^3^]) was estimated on undisturbed samples extracted by a special soil auger with a 100 cm^3^ nozzle. After a standard 12 h drying at 105 °C to a constant mass (*m_s_*, [g]), the bulk density was calculated as ρ*_b_* = *m_s_*/100. The water storage (*WS*, [mm]) in the soil layer *h*, [cm], and the water content saturating the soil (*W*_s_, [%]) were calculated using the formulas [20]: *WS* = ρ*_b_Wh*/(10ρ), *W*_s_ = 100(ρ/ρ*_b_* − ρ/ρ*_s_*), where *W*, [%] is the weight content of water, ρ = 1 g/cm^3^ is the density of water, ρ*_s_* is the density of soil particles (2.65 g/cm^3^ for sandy soils and 2.63 g/cm^3^ for loamy Serozem).

Morphometric and weight control of phytomass and yield was accompanied by their quality assessment. As an indicator of lawn quality, the content of total chlorophyll was determined spectrophotometrically in acetone extract, according to [51]. The antipathogenic quality of potato tubers under the influence of protective gel-forming conditioners was assessed by matrix PCR diagnostics of the main potato pathogens. This express analysis was performed by GenBit LLC [52] and the All-Russian Research Institute of Phytopathology [53] using the AriaDNA microarray amplifier (“Lumex” LLC, Russia) and potato DNA pathogen microarrays for the presence of the following bacterial and oomycetic pathogens: *Phytophthora infestans*, *Pectobacterium atroseptumptum*, *subsp. carotovorum*, *Dickeya dianthicola*, *Dickeya solani*, *Clavibacter michiganensis subsp. sepedonicus*, *Ralstonia solanacearum* (for details, see [4]).

Plant growth data in time (*t*, day) were fitted by the standard Verhulst–Pearl logistic model [54]:

(1) $$H=\frac{H_{m}}{1+a\cdot \exp(-r\cdot t)},$$

where *H* [cm] is the measured parameter (height or diameter of the plant), *H_m_* [cm] is its maximum value corresponding to the “environment capacity”, *r* [day^−1^] is the Malthusian growth parameter, and *a* is a constant associated with the initial conditions.

The characteristic time (half-life) for the synthesis of 50% of organic matter (*T*_0.5_) was estimated using the parameters *r* and *a* using the following formula:

(2) $$T_{0.5}=\frac{\ln(a)}{r}$$

Soil respiration was measured in the field by the standard closed-chamber method [44] using an AZ 7752 portable CO_2_ gas analyzer (AZ Instrument Corp., Hong Kong, China) based on NDIR (nondispersive infrared) waveguide technology. The biodegradation of gel-forming soil conditioners in the field was studied by the application method [20] in plastic containers containing a mixture of hydrogels with calcined sand by studying the decrease in the organic carbon content of such mixtures over time. The content of organic carbon (*C*%) in the samples was determined by coulometric titration using the AN-7529 analyzer (Gomel Plant of Measuring Devices, Gomel, Russia). Modeling of biodegradation of gel-forming polymer materials in soil used a standard exponential model [20]:

*C*(*t*) = *C*_0_ exp(−*kt*), (3)

where *C* and *C*_0_ [%] are the initial content and its change over time due to bio-destruction, and *k* [yr^−1^] is a kinetic constant of biodegradation associated with the half-life of the polymer by the following equation [20]:

(4) $$T_{0.5}=\frac{\ln(2)}{k}$$

A more accurate approach used computer numerical simulation of biodegradation depending on temperature controlling. According to [20], temperature has the greatest influence on the rate of biodegradation of hydrogels almost always saturated with water available for microorganisms. A model with a temperature factor looks like:

(5) $$\frac{dC}{dt}=-k(T)\cdot C;\;k(T)=k_{0}Q_{10}{}^{(T-Tm)/10},$$

where *T* [°C] is the temperature and *k*(*T*) is the standard function “*Q*_10_” for the temperature factor of biodegradation with the parameters: *Q*_10_ [dimensionless], *k*_0_ [yr^−1^], *Tm* [°C]; *k*_0_ and *Tm* are the maximum rate of biodegradation and the temperature optimum for microorganisms, respectively. Computer technological modeling of energy and mass transfer in the “soil-gel-plant-atmosphere” system, according to [42], was based on the HYDRUS-1D software [55]. Statistical and mathematical processing of the results, including data approximation by nonlinear thermodynamic models and ANOVA, was carried out using MS Excel, Microsoft Office 2016 (Microsoft, Redmond, DC, USA), R-3.5.3 (RStudio PBC, Boston, MA, USA), and S-Plot 11 (Systat Software GmbH, Erkrath, Germany) computer software.

## 3. Results

### 3.1. Advanced Intelligent Soil Design

An intelligent soil design based on the HYDRUS-1D computer software [55] and the author’s kinetic models of polymer biodegradation in soils [20] was used to determine the optimal doses and depths of the location of gel-forming soil conditioners. Basic input information includes thermodynamic water retention curves, saturated hydraulic conductivity, material half-life, and potential evapotranspiration. These data should be obtained for soil substrates (untreated control) and gel–soil compositions in a technologically and economically feasible range of hydrogel concentrations, usually from 0.05 to 0.3% [2,4,8]. Multivariate numerical simulation with different doses and depths of soil modifiers, selected plant crops, irrigation methods, and norms make it possible to obtain the necessary and sufficient output information to select optimal soil constructions [42]. The basic output information includes water content dynamics, root water consumption, unproductive water losses, and the corresponding risk of leaching for water-soluble agrochemicals or pesticides, as well as the intensity of biodegradation for polymer soil ameliorants.

**Figure 1:**
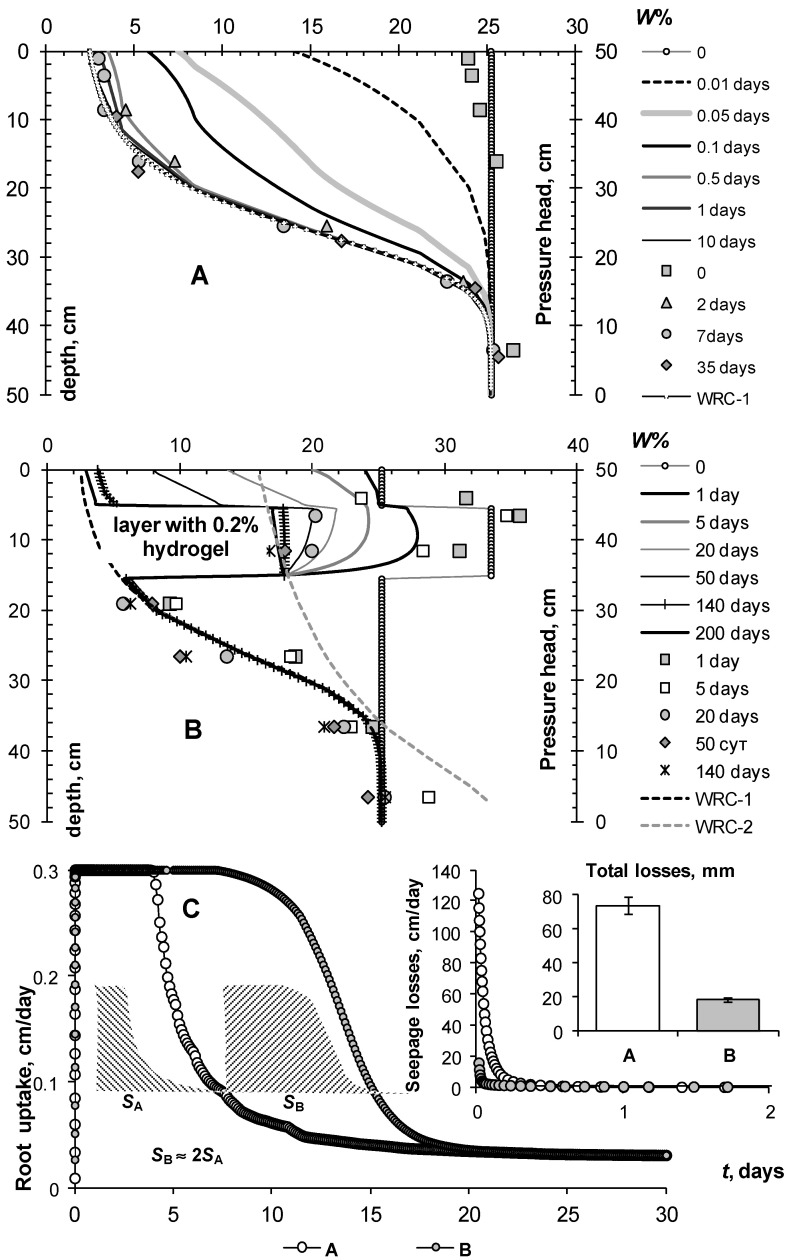
Intelligent soil design using HYDRUS-1D: (**A**)*—*untreated control; (**B**)*—*capillary gel barrier; (**C**)*—*lawn root water uptake (main figure), unproductive water losses (inset); WRC-1,2*—*equilibrium water retention according to WRC of the sandy substrate (1) and its composition with 0.2% hydrogel (2). other lines in figures (**A**,**B**) are the results of water dynamics simulations; symbols are experimental water content data.

Figure 1 represents an intelligent design of soil construction with a capillarity rupture in the subsoil at 50 cm depth (seepage face condition) and with a gel-forming soil conditioner in topsoil. Experimental data to verify the simulation results were obtained in laboratory conditions on models of soil constructions with capillary barriers (for details, see [49]). Using HYDRUS-1D, we simulated the dynamics of water content in soil layers, productive root consumption, and infiltration losses after soil saturation (zero capillary pressure). In the initial sandy substrate with low water retention, water quickly leaves the topsoil, with about 80% nonproductive infiltration losses. Root consumption (grass lawn, roots up to 10 cm) with a potential intensity of 3 mm/day exists for no more than 4–5 days, and then it drops sharply. A small dose of 0.2% hydrogel in a 10 cm layer of topsoil radically improves the water regime of sandy soil. Water is retained in the topsoil; its unproductive losses are reduced by 8 times, and the optimal root consumption is prolonged by 2–2.5 times. The computer dynamics of the water content in the untreated control and under the influence of the gel-forming soil conditioner is in good agreement with the experimental data (symbols in Figure 1A,B).

The total water consumption (the area under the curves in Figure 1C) multiplied by the transpiration coefficient of the tested crop gives its potential productivity, which can be seen to increase up to 2 times relative to the untreated control with a shortage of water in the topsoil.

**Figure 2:**
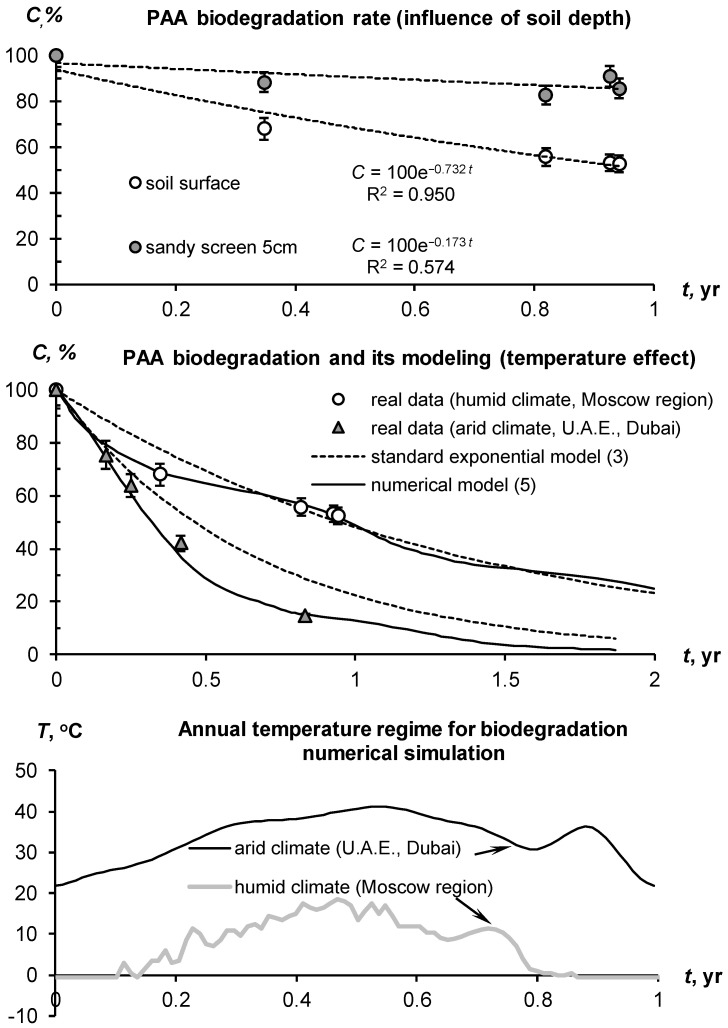
Biodegradation assessment and its computer modeling for PAA hydrogel. Used parameters for the exponential model (3): *k* = 0.732 ± 0.097 (soil surface); *k* = 0.173 ± 0.067 (5 cm depth); Used parameters for the exponential model (3): Parameters used for the numerical model (5): *k*_0_ =1.5 yr^−1^, Q_10_ =2, *Tm* = 20 (humid climate), *Tm* = 30 (arid climate).

Modeling the biodegradation of acrylic hydrogels using PAA superabsorbent as an example shows how quickly a high conditioning effect can disappear if the polymer is located on the soil surface (Figure 2). 

However, shielding the hydrogel with a small layer of sand (5 cm), according to the recommendations [20], allows for a reduction in the biodegradation rate of the acrylic polymer by 2*–*4 times. This theoretical calculation based on nomograms [20] is well confirmed by experimental data (Figure 2). According to the standard exponential model (3), annual losses of the polymer on the soil surface are 100(1 *−* exp(*−*0.732 ± 0.097)) = 52 ± 6%, and under 5 cm of sand 100(1 *−* exp(*−*0.173 ± 0.067)) = 16 ± 7%, i.e.*,* a decrease by 3*–*4 times. A deeper location of the gel-forming soil modifier is impractical both because of the complexity of introducing into the soil and because of the suppression of swelling by pressure from topsoil. For weakly crosslinked PAA superabsorbents, a pressure of 5*–*10 kPa reduces the degree of swelling by 10*–*20 times or more up to small values of 10*–*30 g/g [22,56]. A 30 cm layer of sandy soil with a bulk density of 1.5 g/cm^3^ after saturation by water will exert a pressure of 5*–*6 kPa, which will obviously suppress the swelling of weakly crosslinked superabsorbene at this depth. For the same reason, a more technological method of applying and arranging the hydrogel in a continuous layer (without mixing with the soil) may not be effective, despite a very large (up to 80*–*100 times) decrease in saturated hydraulic conductivity and corresponding infiltration losses, according to [49].

The general results of computer modeling revealed a regular increase in the water supply in the rhizosphere and prolongation of active root water consumption in proportion to the doses of gel-forming soil conditioners. The minimum doses (0.1–0.2%) in a layer of 10 cm is quite enough to increase the available water storage in the rhizosphere by 1.5–2 times compared to the initial sandy substrate and prolong the active (3–5 mm/day) root water consumption from 5 to 10 days. This effect occurs due to an increase in the water retention of gel compositions, as well as due to a strong decrease (up to 10 times) in unproductive losses of water seepage from the rhizosphere with gravitational runoff. Covering the topsoil containing hydrogel by 3–5 cm of native sand ensures the safety of the polymer conditioner from rapid biodegradation and prolongs its service life.

### 3.2. Automated Monitoring of Natural Hydrothermal Conditions

**Figure 3:**
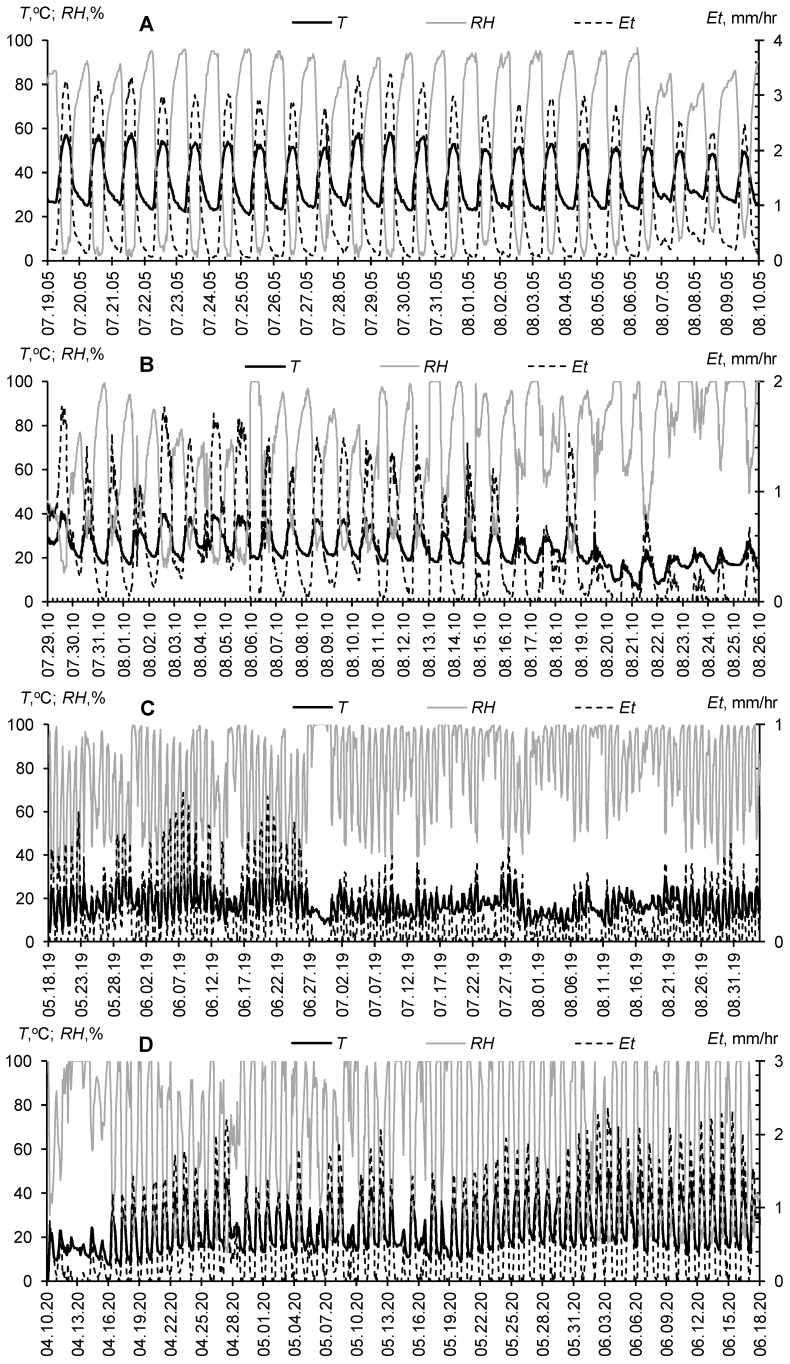
Weather conditions of the growing season (DS1923 monitoring fragments): (**A**)—Qatar (2005); (**B**,**C**)—Moscow region (2010, 2019); (**D**)—Uzbekistan (2020).

**Figure 4:**
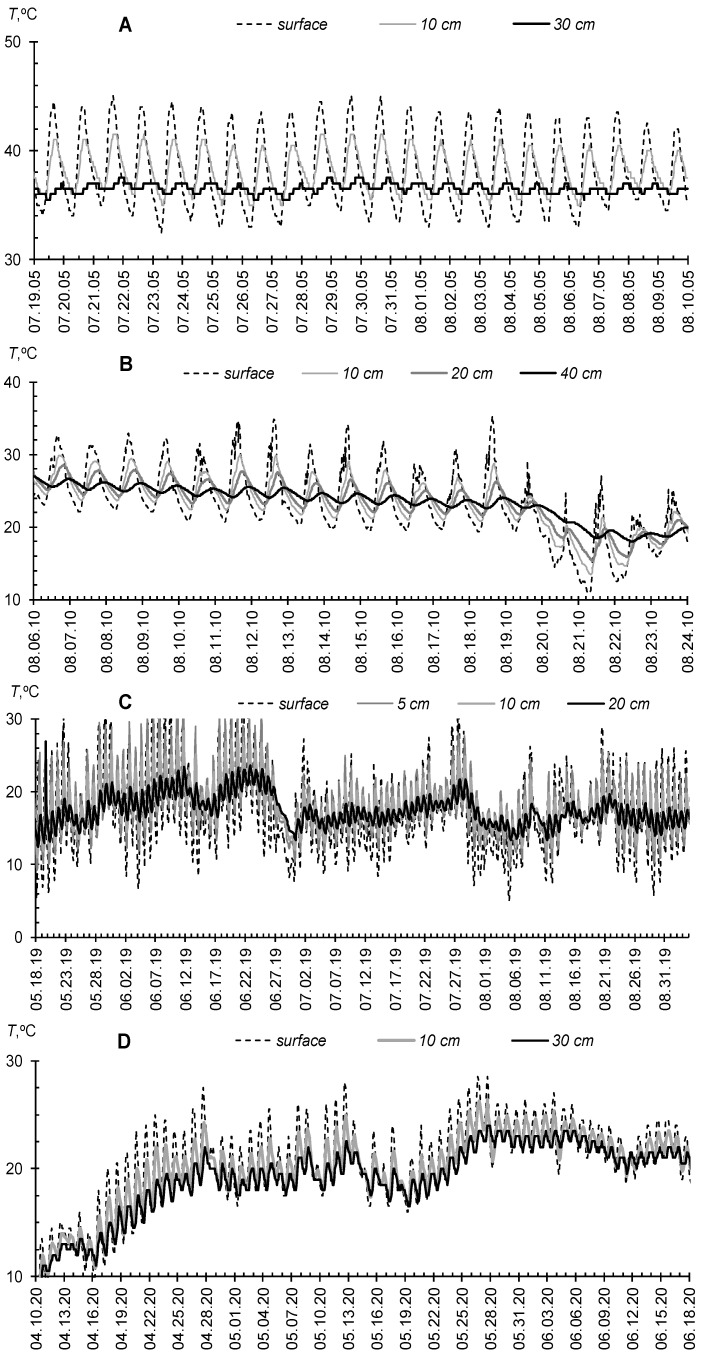
Fragments of soil temperature monitoring (DS1923 loggers): (**A**)—Qatar (2005); (**B**,**C**)—Moscow region (2010, 2019); (**D**)—Uzbekistan (2020).

The greatest effect of gel-forming superabsorbents is expected in arid climatic conditions with an acute shortage of pure water. However, even for the Moscow metropolis in a humid climate, prolonged droughts with high air and soil temperatures are quite possible. Figure 3 and Figure 4 compare hydrothermal conditions in the atmosphere and soil at experimental sites for testing gel-forming soil conditioners in the Gulf (Qatar, Al Utouriya, 2005), Moscow region (2010, 2019) and Uzbekistan (2018, 2020). The potential evapotranspiration (*Et*, [mm/h]) is calculated according to the well-known empirical Ivanov formula [57] with conversion to a 2 h step of measuring temperature (*T*, [°C]) and air humidity (*RH*%): *Et* = 0.0018·(25 + *T*)^2^·(100 − *RH*%)/(30·12). In arid climates (Qatar, Uzbekistan), spring–summer growing seasons are characterized by maximum air temperatures up to 40–50 °C, sharp daily temperature drops (amplitude up to 30 °C), and high potential water evapotranspiration, reaching 1 mm/hour or 12–20 mm/day. In soils, temperature fluctuations are smoothed out, but the average daily temperature values remain very large (25–35 °C). 

For the temperate climate of the Moscow region during extremely dry periods (for example, summer 2010), the air temperature can rise to 40 °C with daily amplitudes of 20–25 °C and potential evapotranspiration values of 10–15 mm/day, which is close to the arid climate. Topsoil warming up to 25–30 °C is also similar to arid conditions, and only in normal seasons with periodic rains; the average daily soil temperatures in summer are within 15–25 °C. Apparently, global climate changes exacerbated by the “heat island” in the metropolis form a trend of convergence of hydrothermal conditions of the studied arid and humid objects. This trend determines the equal importance of soil conditioning with superabsorbents of water in these seemingly different climatic objects.

### 3.3. Lawn Planting in Urban Landscaping with Gel-Forming Soil Conditioners

#### 3.3.1. Experiment in Arid Climate (UAE., Dubai Emirate)

**Figure 5:**
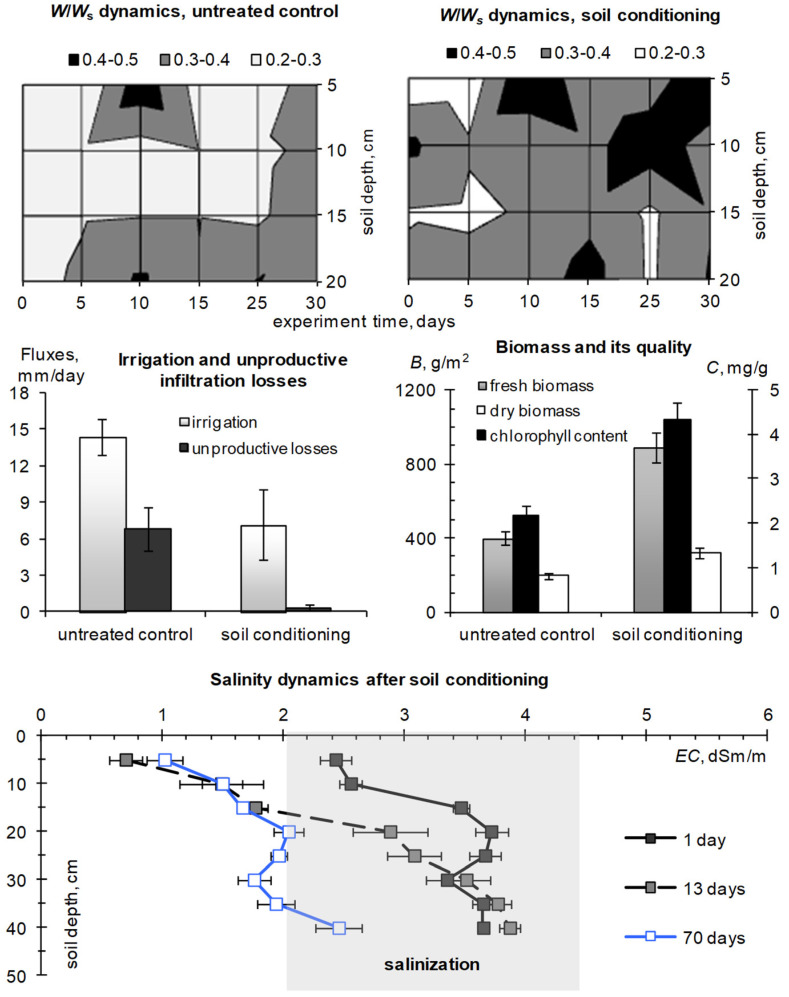
Soil regimes, irrigation, lawn biomass, and its quality under the influence of PAA-gel-forming conditioner (O.A.E. Dubai, 1995).

Our first experiment in 1995 was conducted at the Dubai Municipality, Department of Horticulture and Public Park Station [42]. Instrumental weather monitoring at a similar station in the neighboring Emirate of Qatar provides information about the extremely hot summer season in the Gulf countries (Figure 3 and Figure 4). During the tests of a PAA soil modifier with a small 0.1% dose in a 5–15 cm layer of loamy–sandy Arenosol, we achieved the planned 1.3–2 fold savings in irrigation water by improving water retention and minimizing unproductive water losses (Figure 5).

The *W*/*W_s_* index was within the optimal water content, while for the untreated control it often diagnosed a shortage of water available to plants (*W*/*W_s_* < 0.3). The use of PAA hydrogel reduced unproductive water losses by 10–20 times and increased the phytomass of the lawn by 1.6–2 times, as well as the content of chlorophyll responsible for photosynthesis and visual appearance (dark green color) of plants. The standard electrical conductivity assessment revealed a tendency of topsoil desalinization over 70 days of the experiment due to the hydrogel capillary barrier, according to [49] (Figure 5, lower part). With the cost of PAA hydrogel in 1995 about 6 USD/kg (excluding transfer to O.A.E.) for a working dose of 0.1% in a 10 cm layer, its consumption does not exceed 1.3–1.5 t/ha or, respectively, 8–9 thousand USD/ha. The achieved saving of irrigation water near 6 mm/day or 1800 m^3^/(ha month) at the technological cost of fresh water 2–3 USD/m^3^ in the Gulf is equivalent to 3.6–5.4 thousand USD/(ha month). That is, for two summer months with a high irrigation rate of municipal lawns (12–15 mm/day), the costs of hydrogel are fully paid off by saving scarce water resources.

#### 3.3.2. Experiment in a Temperate Climate (Russian Federation, Moscow Region)

**Figure 6:**
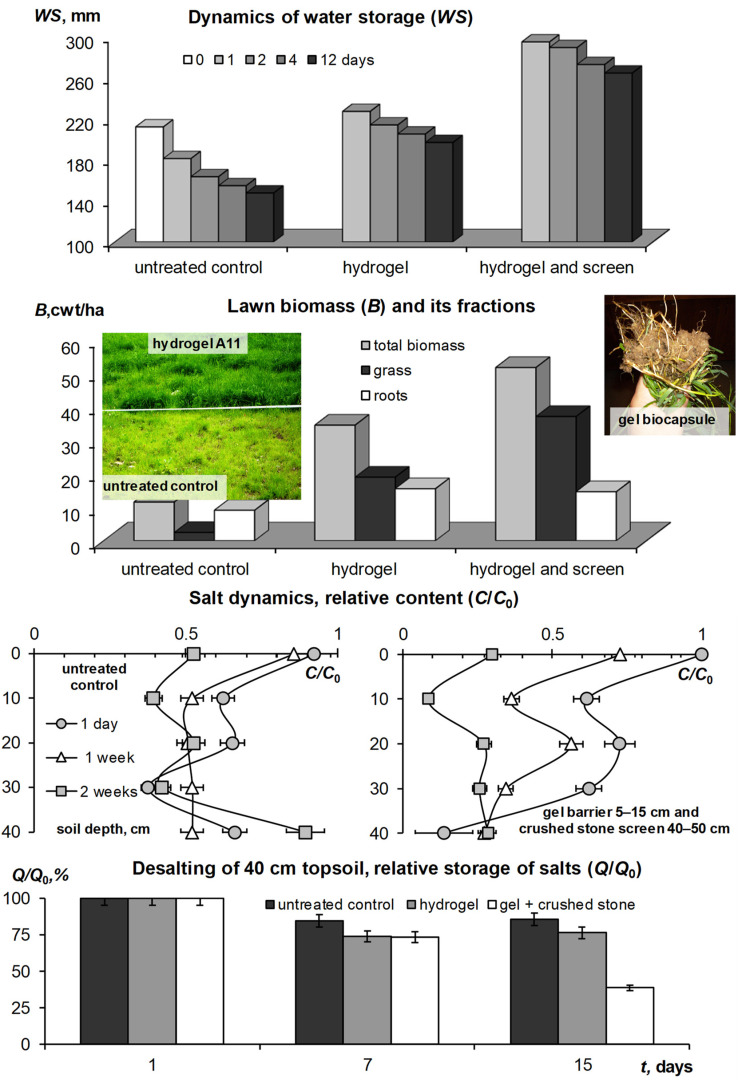
Dynamics of water storage, lawn biomass and topsoil desalinization under the influence of A11-gel-forming conditioner and anti-salt crushed stone screen (Moscow metropolis, 2010).

**Figure 7:**
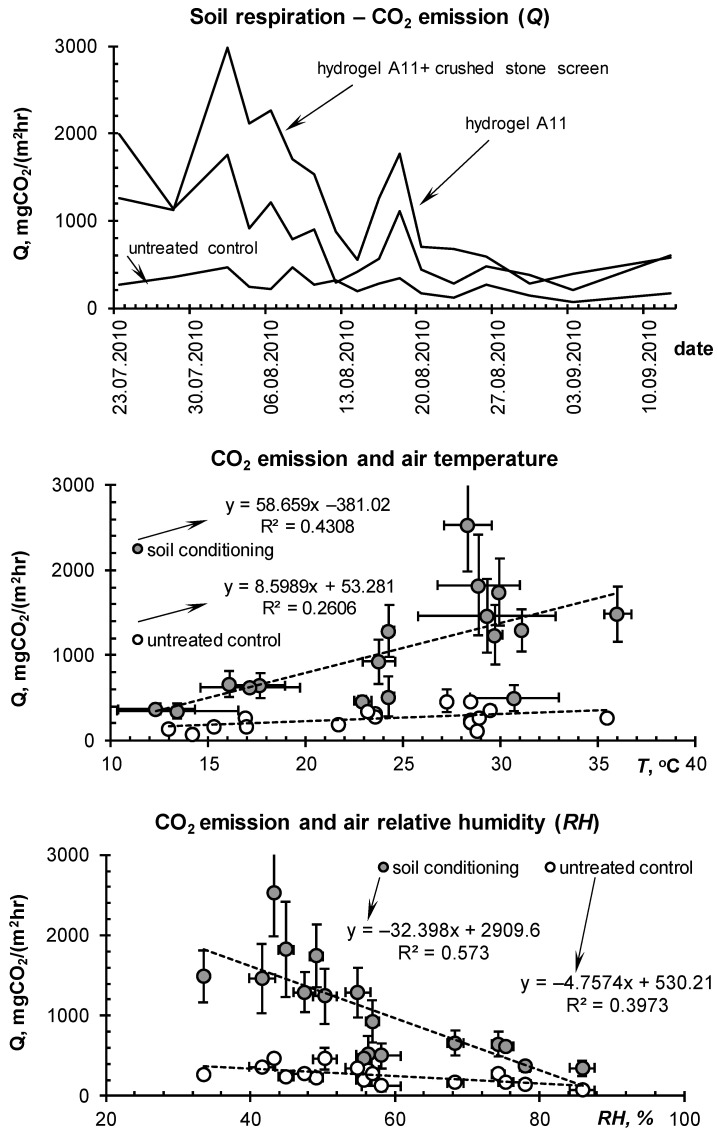
Soil respiration under the influence of A11 hydrogel and controlling factors of temperature and air humidity.

The summer season of 2010 in the Moscow metropolis was extremely hot and was only slightly inferior to the extra-arid climatic conditions of the Gulf in terms of hydrothermal parameters of the atmosphere and soil (Figure 3 and Figure 4). Under these conditions, all the lawn vegetation of the metropolis growing without irrigation was dead [44]. In the tested areas without irrigation, the *W*/*W_s_* index in July dropped below 0.15–0.20, diagnosing the complete absence of water available to plants in topsoil [44,50]. Our technology based on the A11 composite gel-forming conditioner at a dose of 0.2% in a 5–15 cm layer of sandy–loamy Retisol, together with a one-time water-charging irrigation (120 mm), made it possible not only to preserve the green lawn but also to increase its productivity by 2–4 times, due to the corresponding increase in the storage of productive water in topsoil (Figure 6). The dry phytomass of the lawn on the untreated control did not exceed 10–12 cwt/ha with the dominance of roots (80%). For the site with hydrogel, the total dry phytomass reached 35 cwt/ha with close proportions of the aboveground and underground parts (60 and 40%, respectively). The combination of hydrogel with water-accumulative and anti-salt crushed stone barrier further enhanced water retention in topsoil, stopping capillary resorption of water from it into the subsoil, according to [49]. As a result, the storage of productive water and the phytomass of the lawn were maximum here. Dry phytomass exceeded 50 cwt/ha with a proportion of 70% for grass and 30% for roots (Figure 6). The weight and length of the roots, despite a relatively small percentage of the total productivity, were also recorded to be high, reaching 15 cwt/ha and 14 cm, respectively. The roots were closely concentrated in the hydrogel, forming a kind of biocapsule (photo in Figure 6). The hydrogel stimulated the growth of roots and biomass of the lawn as a whole, and also improved its quality in the form of density of the grass cover and intense emerald color of the plants. Along with water retention, the additional supply of nitrogen during the biodegradation of polyacrylamide and ammonium acrylate in the composition of the A11 hydrogel could also have a positive effect. This assumption is indirectly confirmed by the high values of soil respiration in the hydrogel plots compared to the untreated control (Figure 7). Monitoring of this indicator of soil biological activity reveals periodic surges of respiration up to 2000–3000 mgCO_2_/(m^2^·h). Against the background of fairly constant and favorable hydrothermal conditions, such wavelike fluctuations are most likely associated with nonlinear trophic relationships between vegetation and microbiocenosis in the rhizosphere [58]. Microbial surges are accompanied by the biodegradation of plant detritus and other organic materials, including polymer hydrogels, with the entry into solution and root absorption of the biophilic elements necessary for vegetation. Otherwise, soil respiration is significantly controlled up to 50–75% (R^2^ = 0.26–0.57 for linear regressions) by air temperature and humidity (Figure 7, lower part).

The structure with a double capillary barrier in the form of a hydrogel layer (5–15 cm) and a crushed stone screen (40–50 cm) most effectively protected the topsoil from water-soluble pollutants (salinization). In just 15 days of the experiment, the salt concentration decreased by 70–80% in the entire 40 cm soil layer above the gravelly screen (Figure 6, lower part). In the area treated only with hydrogel without an anti-salt screen, a similar desalinization occurred only in the upper 15-cm layer with a gel capillary barrier. For the untreated control, the decrease in salt concentration did not exceed 30–50% with increased accumulation on the surface and in the lower part of the soil profile.

In general, field tests in both arid and temperate climates with summer drought confirmed the high efficiency of gel-forming soil conditioners for urban landscaping with the cultivation of sustainable and highly productive green lawns.

### 3.4. Potato Plantings in Irrigated Agriculture with Gel-Forming Soil Conditioners

#### 3.4.1. Experiments in Arid Climate (Uzbekistan)

Composite gel-forming soil conditioners can be successfully used to optimize edaphic properties and obtain sustainable crops and antipathogenic protection not only in urban landscaping, but also in agriculture [1,2,3,4,5,6,15,33,34,35,36,37,38,39,40,41]. The next field trials of composite hydrogels with combined action (water retention, soil structuring, antipathogenic protection) were carried out in Uzbekistan on potato farms. The arid climatic conditions of Uzbekistan with high temperatures and lack of precipitation in the spring–summer period (Figure 3 and Figure 4) require mandatory irrigation. Traditionally, for Central Asia, flood irrigation by furrows is generally accepted as the easiest way. However, this type of irrigation leads to high (30–50% or more) unproductive water losses through evaporation and infiltration in furrows [6,25]. The introduction of gel-forming soil conditioners into the furrows drastically reduces infiltration losses due to an imperfect capillary barrier, according to [4,49]. Unlike water-soluble PAA, the use of composite, biodegradation-resistant gel structures completely solves the problem of small environmental risks of acrylamide leaching, raised in [6,25].

**Figure 8:**
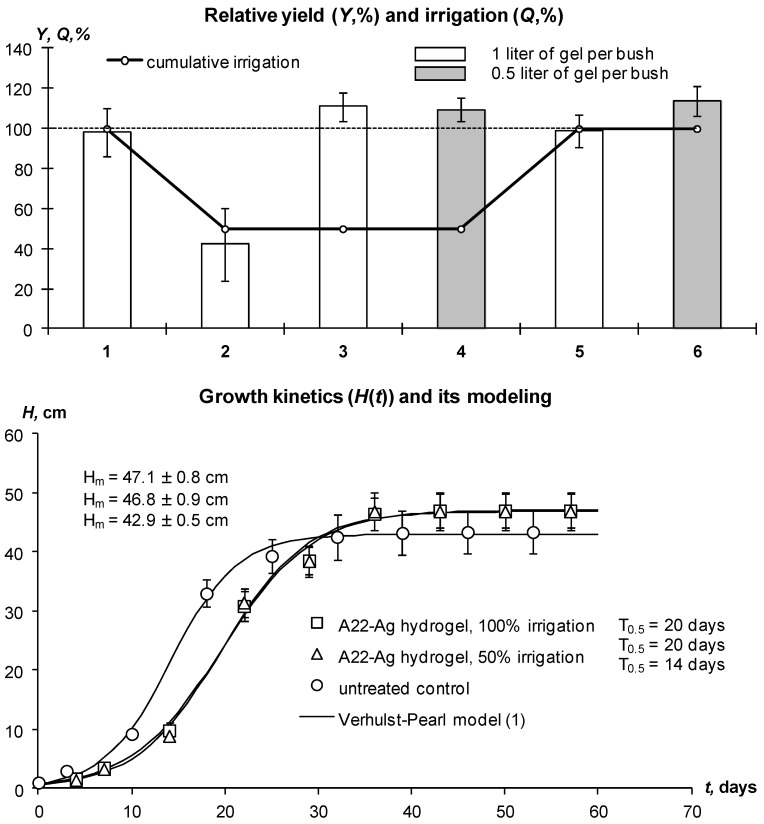
Yield and growth rate for the Gala potato variety under the influence of irrigation and gel-forming soil conditioners (Uzbekistan, Serozem, furrow irrigation, 2018): 1, 2—untreated control, 100 and 50% irrigation norm (400 mm); 3, 4—A22-Ag hydrogel, 50% irrigation; 5—A11 hydrogel, 100% irrigation; 6—A22-Ag hydrogel, 100% irrigation. The dotted line (100%) is the potential yield of 40 t/ha.

In the 2018 experiment, the use of small doses of 2.5–5 g of preswollen A11 and A22-Ag hydrogels reduced irrigation water consumption by 50%, along with maintaining high yields of Gala potatoes (Figure 8). 

At a 100% irrigation rate with a total consumption of 400 mm of water on the untreated control, the average yield reached 98 ± 12% of the planned potential yield for this potato variety (40 t/ha). However, a twofold decrease in irrigation (50% norm) causes a sharp (more than 2 times) decrease in potato yield with an increase in its variation (42 ± 18%). The use of gel-forming soil conditioners statistically significantly increased the yield by 12–15% relative to untreated control, as well as the height of the potato bushes, size, weight, and in some cases the density of the tubers, which could be caused by the accumulation of starch. The growth of the Gala potato variety was in good agreement with the standard Verhulst–Pearl logistic model (1) [54] with parameters of *r* = 0.21–0.35 day^−1^, *T*_0.5_ = 14–21 days, *H_m_* = 43–47 cm statistically significant with a *p*-value of 0.001–0.014 (Figure 8, lower part). Large doses of hydrogels (1 L per bush) slowed down the germination of tubers and the rate of further growth of potatoes with a *T*_0.5_ difference of 3–6 days. The plots treated with hydrogels at 100% and 50% watering did not statistically significantly differ from each other in yield (Figure 8); therefore, saving irrigation water by 50% in the case of using hydrogels does not lead to a decrease in potato yield.

**Figure 9:**
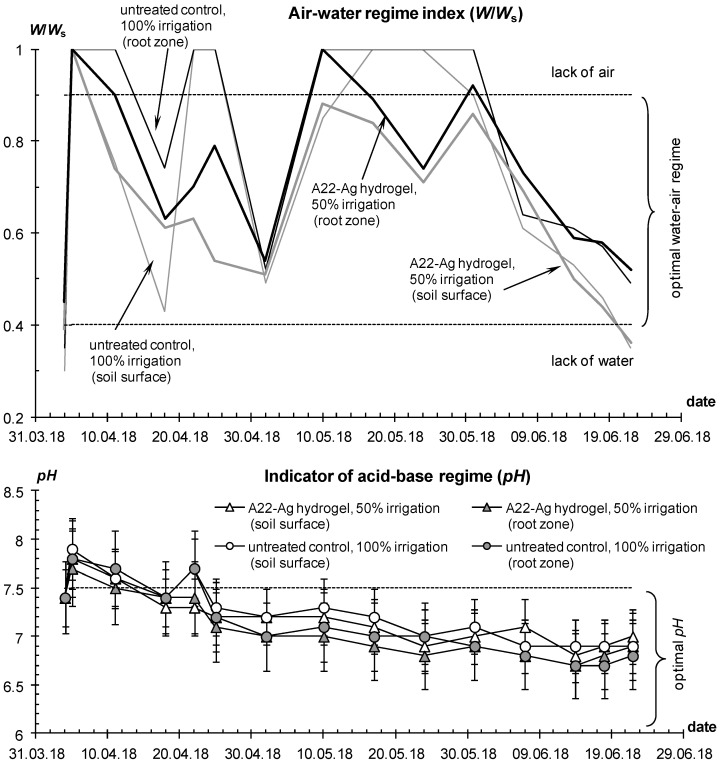
Soil regimes under the influence of irrigation and gel-forming conditioners (Uzbekistan, Serozem, furrow irrigation, 2018).

The water–air regime index *W*/*W_s_* for the untreated control often diagnosed the state of waterlogging, while in plots with hydrogels mixed with soil at doses of 0.5 L/plant, the W/Ws value was more often in the optimal range according to the concept of soil physical quality [50], not exceeding 0.9 (lack of air) and not falling below 0.4 (lack of water available to plants) (Figure 9). 

At a high dose of 1 L per bush and 100% irrigation, waterlogging of the root zone (*W*/*W_s_* >0.9) often occurred; however, the tubers did not rot due to strong potential evaporation and high transpiration activity of plants. Treatment with hydrogels stimulated the biological activity of the soil and lowered the *pH* relative to the values in the untreated control (Figure 9, lower part). At the beginning of the season, *pH* exceeded 7.5 or the lower limit of alkaline inhibition for a number of potato varieties, but then, during the growing season, the *pH* of the rhizosphere decreased to 6.7–7, which was favorable and ensured the mobilization of mineral nutrition elements. The general tendency of the *pH* decrease during the growing season is explained by the accumulation of biogenic CO_2_ due to plant growth and development (root respiration), as well as the biodestructive activity of soil microflora. This trend was also confirmed by the acceleration of soil respiration from 250–330 mg CO_2_/(m^2^ h) at the beginning of the season to 400–540 at the control and up to 520–740 mg CO_2_/(m^2^ h) on plots with hydrogel compositions. The salt regime changed insignificantly during the season, and the electrical conductivity index in soils in the state of saturation with distilled water did not exceed 3.6–4 dS/m, i.e., the limits of the absence of salinity for soils in the arid zone, according to soil standards [44,48].

Matrix PCR diagnostics did not reveal potato pathogens in tubers protected by biocidal gel structures with silver (composite material A22-Ag), while in the untreated control, dangerous potato pathogens were found sporadically (up to 5% of the examined tubers): *Pectobacterium carotovorum subsp.* ((Jones, 1901) and *Pectobacterium atrosepticum* (van Hall, 1902), causing potato blackleg disease, as well as late blight (oomycete *Phytophthora infestans* (Mont.) de Bary). These pathogens can cause great damage to potato growing in Uzbekistan, for example, in the Tashkent region, where according to [59], the degree of damage to the crop only by late blight has reached 19–36% since 2015. In this regard, the use of gel-forming soil conditioners with a biocidal effect that protects the rhizosphere from major pathogens is relevant for irrigation potato farming in Uzbekistan. A rough economic assessment of the yield increase gave a 1.5–2-fold payback for the most effective composite, A22-Ag based on amphiphilic gel with 50 ppm ionic silver at a dose of 2.5–4 g of dry gel per bush, calculated at the purchase price in the Russian Federation and in Belarus in 2018 for marketable potatoes about 0.10–0.13 USD/kg. That is, in intensive irrigated agriculture, this technology is quite acceptable not only for seed potato growing with high prices for elite potatoes, but also for profitable commercial production.

**Figure 10:**
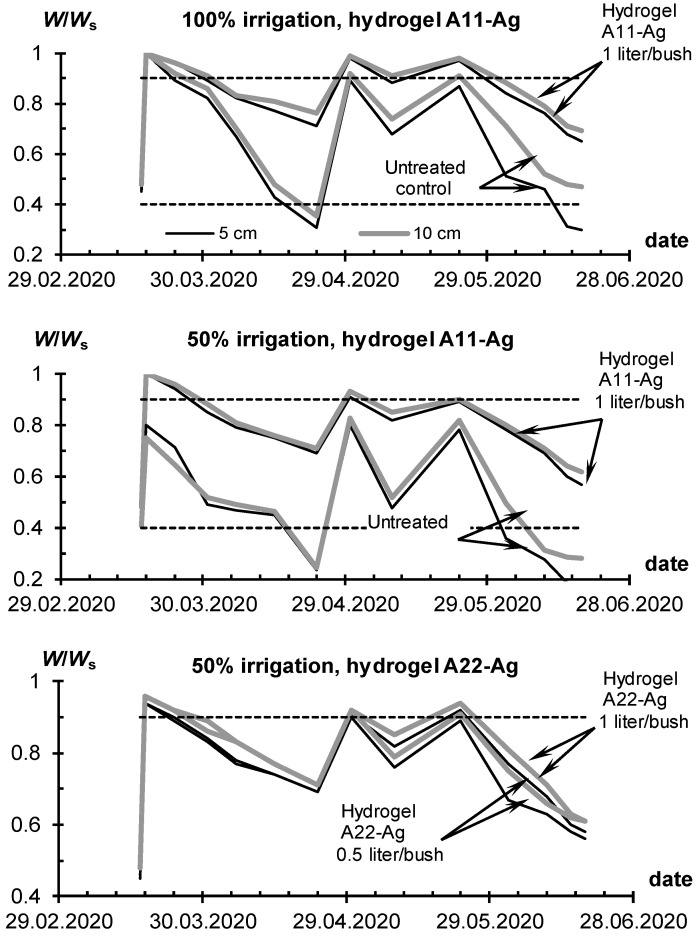
Soil water–air regime under the influence of irrigation and gel-forming conditioners (Uzbekistan, Serozem, furrow irrigation, 2020). The dotted lines are the critical limits of the optimal values for the *W*/*W*_s_ index (see Figure 9).

Similar results were obtained in a repeated field experiment with the Santana potato variety, conducted in the same farm in 2020. However, due to COVID-19 restrictions, only three flood irrigations were carried out here instead of the usual four. The dynamics of the index of water–air regime (Figure 10) clearly reflects the irrigation of three bursts of humidity and a long (more than a month) non-irrigated period until the beginning of May. During these periods, the *W*/*W_s_* index briefly exceeded the upper limit of 0.9, diagnosing a problem with soil aeration [50]. However, this situation, the same as in the experiments of 2018, could not cause much damage to vegetation due to the short duration, as well as the high rate of evapotranspiration (1–2 mm/h, according to Figure 3D). Excluding these bursts, the untreated control experienced intermittent water deficiency (*W*/*W_s_* < 0.4), especially at a 50% irrigation rate. Plots with gel conditioners consistently demonstrated higher *W*/*W_s_* values compared to the untreated control due to the higher water-holding capacity of the gel–soil compositions. Reducing the dose of hydrogel from 1 to 0.5 L per bush had little effect on the water regime of the soil. The *W*/*W_s_* index on plots with a low dose of 0.5 L per bush remained in the optimal range from 0.4 to 0.9 during the entire time of the experiment, even with a 50% reduction in watering rate (Figure 10). This result, as in the previous experiment in 2018, confirmed the possibility of a double reduction in the hydrogel dose and irrigation rate without significant damage to productivity. The water-retaining effect of two composite hydrogels with hydrophilic (A11) and amphiphilic (A22) fillers did not differ significantly for the same application doses.

**Figure 11:**
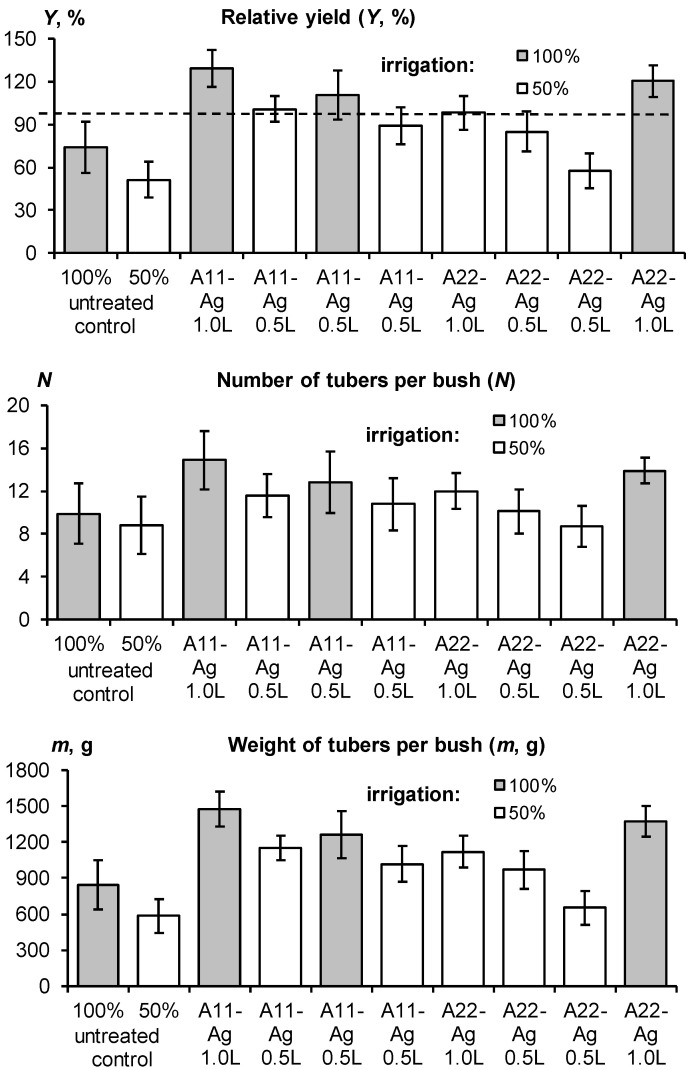
Yield and its components for the Santana potato variety under the influence of irrigation and gel-forming soil conditioners (Uzbekistan, serozem, furrow irrigation, 2020). The dotted line (100%) is the potential yield of 40 t/ha.

The lack of water adversely affected the yield of the control plots and emphasized the advantage of treatment with gel-forming soil conditioners (Figure 11). For the untreated control at 100% irrigation rate, the average potato yield was 74 ± 18% of the potential, and in the case of 50% irrigation water saving, it was only 51 ± 13%. On plots with gel-forming conditioners, the potential yield of 40 t/ha for the variety “Santana” was achieved, and even exceeded in the case of gel application at a rate of 1 L/bush at 100% irrigation. Economical doses of gel of 0.5 L/plant and/or water saving (50% of watering) left the yield at the level of potential or 30–60% more than for the untreated control. Regular differences between untreated and conditioned plots arose not only in terms of the total potato yield, but also in terms of its individual indicators—the number of tubers from one average bush and their weight (Figure 10). Both indicators increased significantly in the case of using hydrogels at 100% irrigation rate. Hence, gel-forming soil conditioners have a twofold positive effect on both the number and weight of tubers. As in the previous experiment in 2018, diagnostics of pathogens did not reveal a potential infection of tubers in variants with protective gel structures, while pathogens *Pectobacterium atrosepticum* and *Pectobacterium carotovorum subsp.*, apparently characteristic of these conditions, were present in the untreated control.

#### 3.4.2. Experiments in a Greenhouse with Drip Irrigation (Russian Federation, Moscow Region)

**Figure 12:**
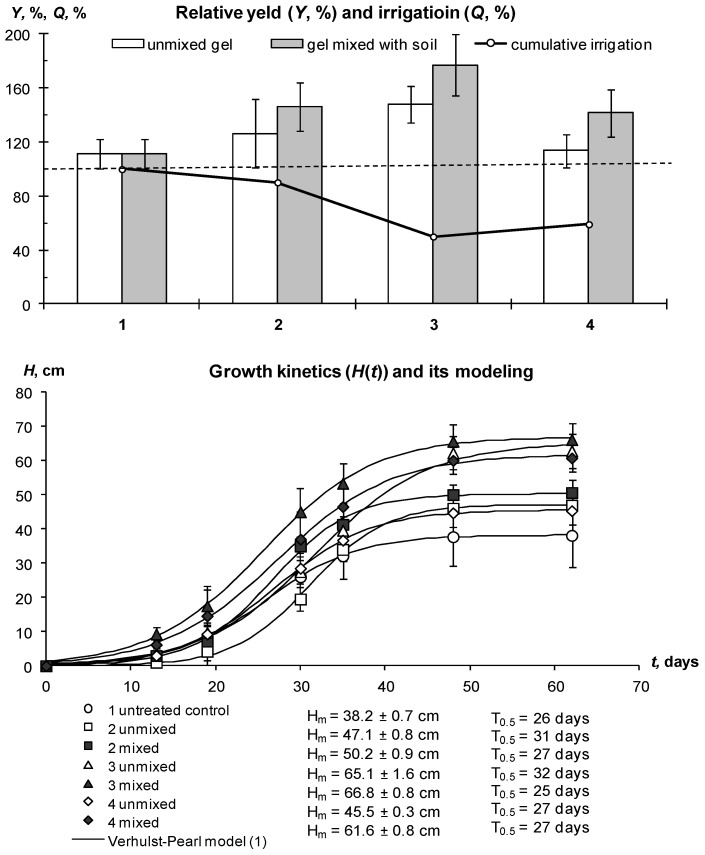
Yield and growth rate of the Red Scarlett potato variety under the influence of irrigation and gel-forming soil conditioners (Moscow region, Retisol, drip irrigation, 2018): 1—untreated control; 2—A22 + Quadris 50 ppm; 3—A22 + Ag ions 100 ppm; 4—A22 + Ag nanoparticles 100 ppm. Biocides are introduced into the solution for preswelling of hydrogels before application in the soil. The dotted line (100%) is the potential yield of 30 t/ha.

Additional experiments carried out in closed ground (ventilated greenhouse) with drip irrigation in the conditions of the Moscow region also allowed us to achieve—and even exceed—the potential yield of the tested Red Scarlett potato variety of 30 t/ha with 30–50% savings in irrigation water. The first experiment in 2018 tested gel structures with silver ions and nanoparticles, as well as the organic fungicide Quadris as part of an intermicellar solution at concentrations of 50–100 ppm. The average yield at all sites was higher than the potential; however, a statistically significant (*p* ≤ 0.05) excess was achieved only on plots treated with gel-forming soil conditioners (Figure 12). The best results (30% excess of potential yield and more) were demonstrated by composition A22 with an amphiphilic filler in the form of dispersed peat and with silver ions. The untreated control had a minimum average yield that exceeded the potential yield by 10%, but not statistically significantly. Compositions with silver nanoparticles and the fungicide Quadris occupied an intermediate position.

Mixing the hydrogel with the soil increased the average yield on the treated plots by up to 20% or 6 t/ha; however, this increase was not significant at the accepted *p*-value level (*p* ≤ 0.05) due to the high variation in the data (confidence intervals of 5–9 t/ha). Both the tuber yield and the aboveground potato phytomass in the treated plots were higher by 20–50% compared to the untreated control. However, the growth kinetics of potatoes was similar in hydrogel-treated and nontreated plots (Figure 12, lower part). It was well fitted by the standard Verhulst–Pearl model (R^2^ = 0.997–0.999) with *H*_m_ from 38 to 67 cm and *r* from 0.15 to 0.22 day^−1^, statistically significant at *p*-level 0.001–0.037. The half-life index (*T*_0.5_) calculated by the formula (2) varied from 26 to 32 days and did not explicitly depend on treatment, in contrast to previous experiments on the loamy soil of Uzbekistan. It appears that better aeration in sandy soils does not cause growth retardation under treatment with gel-forming conditioners observed in loamy soils with less air permeability.

**Figure 13:**
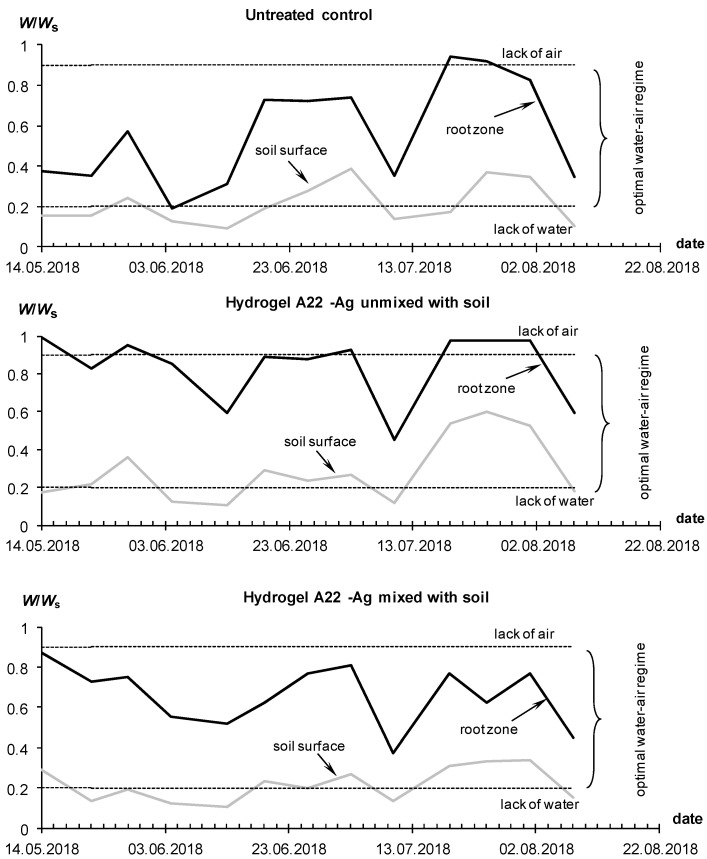
Soil water–air regime under the influence of irrigation and gel-forming conditioners (Moscow region, Retisol, drip irrigation, 2018). The dotted lines are the critical limits of the optimal values for the *W*/*W*_s_ index (see Figure 9).

Due to the fully automated drip irrigation system with the ability to adjust the supplied water through timers, it was possible to reduce water consumption for all plots with hydrogels, and in economical irrigation, this reduction reached 1.7–2-fold differences. That is, saving up to 40–50% of irrigation water not only did not damage productivity, but even increased the potato yield. This phenomenon is explained by high (50–80% or more) unproductive losses of irrigation water in coarse-textured soils due to subsoil infiltration and evaporation, and these losses are almost completely blocked by local gel structures in the rhizosphere under drip irrigation of each potato bush. Matrix PCR diagnostics did not reveal pathogenic organisms either in the control or in the variants with hydrogels, which can be explained by the good quality of the seed material and the dry conditions of the ventilated greenhouse unfavorable for pathogenic microflora. Comparative monitoring of the water–air regime index showed that it was mainly within the optimal zone for this soil of 0.2 < *W*/*W_s_* < 0.9, especially for the root layer, and only in the variants of continuous (without mixing with soil) hydrogel application occasionally exceeded this range (Figure 13). The *pH* did not cross the critical level of 7.5, was consistently lower in the rhizosphere compared to the soil surface, and tended to decrease down to 5.6–6.5 during plant development due to the intensification of soil respiration (acidification with carbon dioxide).

**Figure 14:**
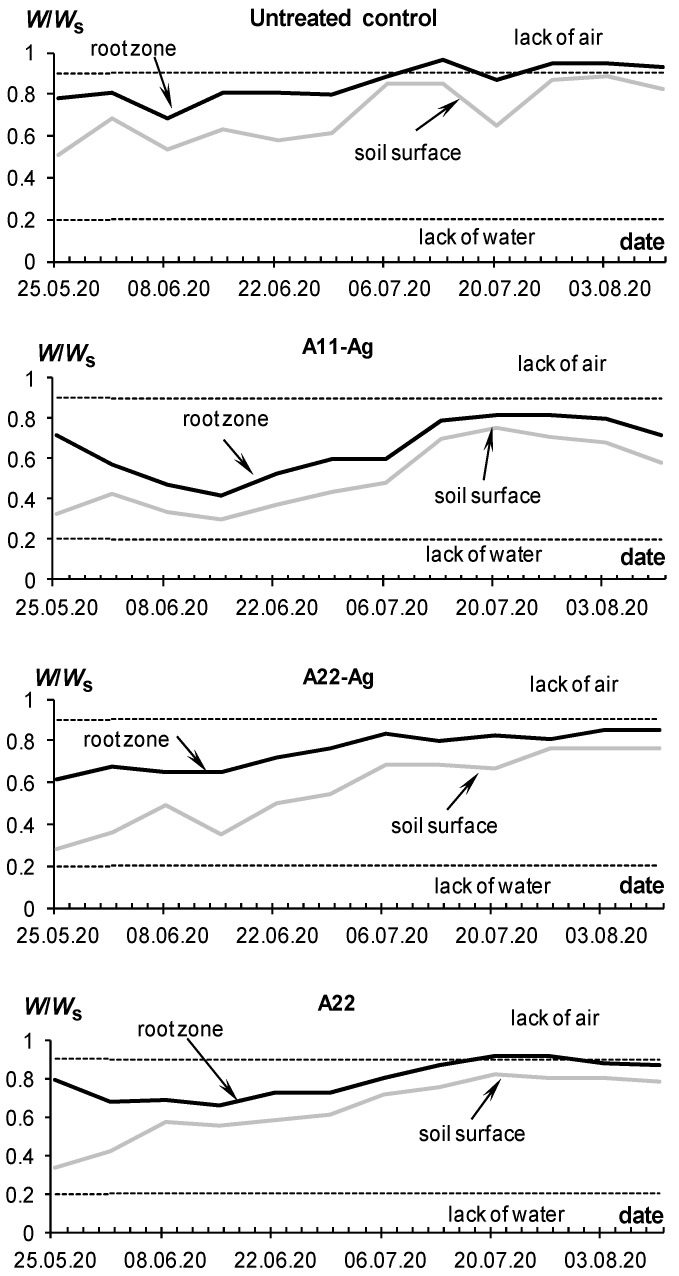
Soil water–air regime under the influence of irrigation and gel-forming conditioners (Moscow region, Retisol, drip irrigation, 2020). The dotted lines are the critical limits of the optimal values for the *W*/*W*_s_ index (see Figure 9).

**Figure 15:**
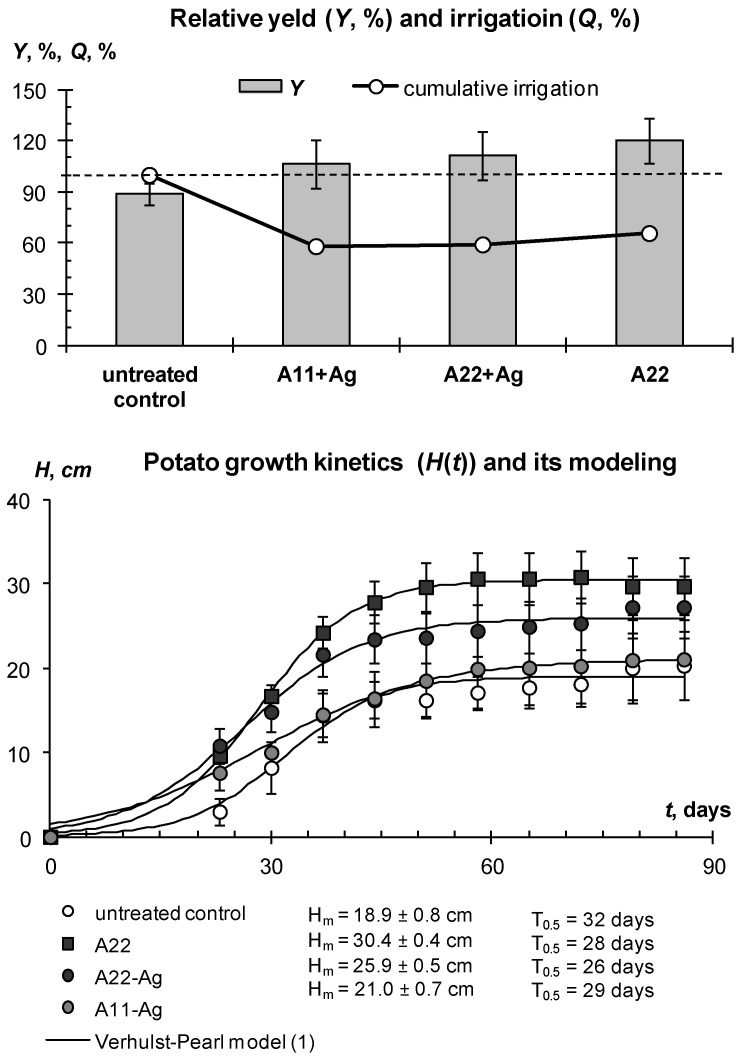
Yield and growth the Red Scarlett potato variety under the influence of irrigation and gel-forming soil conditioners (Moscow region, Retisol, drip irrigation, 2020). The dotted line (100%) is the potential yield of 30 t/ha.

The same results were obtained in a final greenhouse trial using the new gel-forming soil conditioners with silver incorporated directly into the polymer matrix (Figure 14 and Figure 15). On experimental sites with hydrogels during the growing season, the *W*/*W_s_* index was within the optimal zone. Taking into account the results of previous experiments, we used here only the treatment of mixing gel and soil. This method, on the one hand, allows us to improve the aeration of soil and seed tubers at the initial stages of germination, and on the other hand guarantees an even distribution of the protective antipathogenic composition throughout the entire volume of the rhizosphere. Despite a 35–50% reduction in the irrigation rate (286 mm per season) on the treated plots, the water content in the root zone did not fall below the critical level (*W*/*W_s_* = 0.2) due to the water-retaining effect of gel-forming soil conditioners. The *W*/*W_s_* index for the untreated control was generally higher due to the 100% irrigation rate and possibly the lower transpiration activity of less developed potato bushes (Figure 14). 

By the end of the season, the water consumption of the plants decreased, which led to an increase in water content up to a critical level of a lack of soil aeration (*W*/*W_s_* = 0.9), according to the concept of physical soil quality [50]. However, this fact could not cause damage to the crop due to the shortness of time (less than a week), and most importantly, locality, due to drip irrigation techniques.

The growth of aboveground phytomass was characterized by kinetic parameters close to the previous experiment in 2018 (*T*_0.5_ = 23–32 days), but much lower maximum heights of bushes (Figure 15, lower part). It is difficult to explain such differences for the same potato variety and type of irrigation; however, a possible reason was the later planting dates (end of May 2020 instead of mid-May in 2018). The Verhulst–Pearl standard model (1) also successfully fitted the experimental data of potato growth kinetics (R^2^ = 0.957–0.998) with parameters *r* = 0.12–0.15 day^−1^, *H*_m_ = 19–30 cm, statistically significant at *p*-value 0.001–0.004. Despite the lower aboveground phytomass, the tuber yield in 2020 was close to that of 2018 (Figure 12 and Figure 15). The yield of the untreated control was 10% (3 t/ha) lower than potential, while the treatment with gel-forming soil conditioners led to an Increase in yield up to 20–35% (6–9 t/ha) relative to potential (30 t/ha) and to the average reference yield on the untreated control (26.7 ± 2.0 t/ha). A large yield variation (confidence interval 6–14% or 2–4 t/ha) did not allow for statistically significant differences between various gel-forming conditioners, excluding composition A22. Here, the yield was 20% higher than the potential one at the accepted significance level, *p* = 0.05.

Visual inspection of the tubers and subsequent PCR diagnostics did not reveal the most dangerous potato pathogens in the plots treated with silver hydrogels, while the presence of pathogens (*Phytophthora infestans, Pectobacterium atrosepticum, Clavibacter michiganensis subsp. sepedonicus*) was detected on the untreated control and partially in the A22 treatment without biocides. During the storage of the crop indoors at an air humidity of 42–50% and room temperature (18–22 °C) for 3 months, we found rotting of 10–12% of the tubers of the control plots and 5–7% from the A22 GHS site. During the 3 month storage of the potatoes indoors at an air humidity of 42–50% and room temperature (18–22 °C), rotting of 10–12% of the tubers of the control plots and 5–7% of the tubers from the site treated with hydrogel A22 were detected. The potato harvest nets from the plots protected by new gel compositions with silver were in good condition, with no signs of tuber rot. These results confirm the effectiveness of new gel-forming soil conditioners with embedded silver no less than in the case of biocides being introduced into intermicellar solutions of pure hydrogels. This means that the biocides in the polymer matrix of the swelling gel, preserving it from biodegradation and being more reliably fixed in its composition, simultaneously inhibit the development of pathogenic microflora after hydrogels mixing with the mineral mass of the soil.

## 4. Discussion

Our results from field trials of gel-forming soil conditioners were in good agreement with known published data [31,32,33,34,35,36,37,38,39,40,41]. Abd El Aziz et al. [40] showed that an ecofriendly hydrogel based on starch and pectin in greenhouse experiments with tomatoes allowed for a reduction in irrigation by 25–50%, increasing soil water retention by 2–2.8 times. Koupai and Asadkazemi [32] reported a 44% reduction in irrigation without damage to the growth of an ornamental plant (*Cupressus arizonica*) under the influence of superabsorbent A200 at doses of 0.4–0.6%. In a comprehensive 3-year field study [41], soybean and wheat yields increased by 11–18% with 100% irrigation under the influence of Pusa composite hydrogel and its varieties based on cellulose and kaolin. In the case of economical irrigation and in rainfed areas, a similar increase in yield under the influence of hydrogels reached 21–53% compared with the untreated control. Various researchers have reported yield increases of 12–31% in rice [35], 31–46% in maize and soybean [33,34], 5–18% in wheat and oat [31,37,38,41], and 10–25% in oilseed grasses [36,41] under the influence of the gel-forming soil conditioners. Our result on the positive effect of hydrogels not only on the aboveground phytomass and yield, but also on the roots, is confirmed by an independent study [39]. The possible delay in plant growth under the influence of hydrogels, obtained here in some of our experiments with potatoes, is also reported by [41] on the example of wheat cultivation. The benefit of fixing pesticides and agrochemicals in gel formulations is shown in [3,4,5,15,37,60]. In [60], the hydrophobic filler improves the quality of the composite system with the controlled release of pesticides, which partially explains the better protective properties and higher yield effect from our amphiphilic composition A22 compared to hydrophilic analogues.

Our field experiments and modeling of the decomposition rate of polymer gel-forming materials in the soil refute the popular opinion about their high resistance to biodegradation. Lentz et al. [6] reports a rather slow decomposition of PAA not more than 10% per year primarily through the shear-induced chain scission and photodegradation. In the review [30], stable “non-biodegradable” synthetic hydrogels are contrasted with biodegradable ones based on natural biopolymers, mainly polysaccharides. At the same time, publications [25,26,27,28,29] report high biodegradability of synthetic acrylic gels and their copolymers. Sojka and Entry [29] found that PAA was completely degraded within 5 days after applying 0.05% to garden soil. Lande et al. [25] estimated the half-life of acrylamide monomer in agricultural soils ranged from 18 to 100 h at a concentration of 25–500 mg kg^−1^ and a temperature of 20–22 °C. Soil microorganisms are capable of utilizing PAM or acrylamide as a source of nitrogen [26,28,29]. The review [61] also contrasts the traditional opinion in polymer chemistry about the stability of agricultural crosslinked superabsorbents with an extended service time of 5–7 years with numerous data on their rapid biodegradation by soil microorganisms, including fungi. All these facts indicate a low potential stability of acrylic hydrogels in soils and the dominant mechanism of their biological (biochemical) degradation, rather than chemical or photochemical decomposition. In this regard, the development of more stable composite hydrogels with biocidal additives, as well as intelligent soil design that selects optimal depths for hydrogels in the soil in order to reduce biodegradation, seem to be quite relevant directions for solving this problem. The introduction of ionic silver directly into the acrylic polymer matrix, as shown in [8], significantly (up to 15–20 times) increases the half-life of the composite material, and accordingly, its resistance to biodegradation in the soil. The use of silver nanoparticles is more problematic due to the low coagulation threshold in colloidal solutions without special stabilizing agents [47]. Therefore, it is better to add silver nanoparticles to the intermicellar solution directly during the preparation of gel structures for soil application [4]. Silver is a highly effective universal biocide; however, the use of antimony or copper in relation to the fungicidal protection of soil by gel-forming composites may also be promising [62]. At the same time, the use of salts of heavy metals as biocides is accompanied by the risk of contamination of soil and groundwater after the biodegradation of the gel carrier. A more environmentally friendly alternative is to use synthetic fungicides with a low half-life inside a gel-forming composite with a high half-life, i.e., more resistant to biodegradation [4]. In this case, the fungicide will decompose faster than the gel carrier, which guarantees its environmental safety. This circumstance allows us to consider synthetic gel-forming composites more suitable for carrying pesticides, compared to the traditionally used easily biodegradable biopolymer carriers, for example, polysaccharides [5,15].

Another serious technological problem is the kinetics of swelling of soil gel-forming conditioners, as a factor limiting the effectiveness of the delay (interception) of irrigation water in the topsoil. Shahid et al. [12] and our studies [8] show a rather slow swelling rate with the half-life parameters *T_0_*_.5_ = 5–8 h, according to the standard exponential model of first-order kinetics [8,60]. A faster swelling of acrylic superabsorbents with half-life *T_0_*_.5_ = 0.5–1 h was observed by Shirinov and Jalilov [63]. However, as the authors [22,56] correctly note, the potential free swelling slows down sharply in the limited pore space of the soil and under the action of external lithological pressure. Recent data from Louf et al. [64] confirm the long time (50 h or more) of swelling for hydrogels under confining pressure of 0.2–22 kPa. The generally accepted methodological approach to solving this problem is to influence the controlling factors such as crosslinking density, *pH*, hydrogel granule size, etc. [60,65]. We believe an alternative and simpler solution is to introduce not dry or granular but preswollen hydrogels into the soil [4,8]. This method greatly (from 10 to 100 times) reduces the saturated hydraulic conductivity of the soil [8] and guarantees uniform distribution of gel-forming soil conditioners in the soil in small doses (0.1–0.3%).

The most debatable, from our point of view, is the question of the application doses of gel-forming soil conditioners necessary and sufficient to obtain the effects discussed above. In our experiments, the maximum hydrogel consumption was in the case of lawns for urban landscaping. Both computer soil design data and subsequent field trials confirmed the effect of doses of 0.1–0.2% (wt) in a layer of at least 10 cm. These doses in terms of area obviously give at least 1–2 tons of dry hydrogels per hectare in the case of continuous (over the entire area) application. For local application (in furrows or under a potato bush), the hydrogel consumption can be significantly reduced. For example, an effective dose of gel swollen from 1:100 to 1:200 is 0.5 L/bush or 2–5 g of dry hydrogel per bush at a potato planting density of 40–60 thousand per bushes gives a consumption of dry hydrogel from 100 to 300 kg/ha. Similar application rates from 60 to 225 kg/ha can be found in [34,37]. However, Rajanna et al. [41] consider such a consumption of dry gel-forming materials too large and economically unprofitable. These authors report a new semisynthetic cellulose derivative-based product (Pusa Jal Nidhi trademark) capable of increasing the water content in the soil by 3–5% and the biomass yield of soybeans and wheat by 9.5–54% in doses of only 2.5–5 kg/ha. From our point of view, this is simply impossible, unless the hydrogel is transformed into a super-thin film that interrupts the infiltration of water in the soil. A dose of 2.5–5 kg/ha is equivalent to 0.25–0.5 g/m^2^. This small amount of dry polymer is practically impossible to uniformly distribute in the soil over an area of 1 m^2^. With a maximum degree of swelling of 800 g/gm [41], the Pusa hydrogel applied at this dose can retain only 200–400 g of water per 1 m^2^ of soil, or 0.2–0.4 mm of precipitation (irrigation water). In order to increase the water content by 3–5% in a 10 cm soil layer at a bulk soil density of 1.3 g/cm^3^, at least 4–6 L of water per m^2^ are required, which means 15–20 times higher doses of hydrogels. These simple calculations obviously disprove the possibility of effective use of gel-forming soil conditioners for water retention in such small doses as in [41].

## 5. Conclusions

Intelligent soil design based on a computer model of energy and mass transfer in the “soil-gel-plant-atmosphere” system predicts a 1.5–2-fold increase in water storage in the rhizosphere, a 10-fold reduction in nonproductive water losses, a 30–50% prolongation of root water consumption, and a corresponding increase in phytoproductivity under the application of 0.1–0.2% (wt) gel-forming soil conditioners in a 10 cm layer of topsoil. Covering the treated soil with a 3–5 cm layer of sand protects polymer hydrogels from rapid biodegradation, prolonging their service life as a soil conditioner by 2–4 times. These results of computer technological modeling were completely confirmed by direct field trials of composite gel-forming soil conditioners for irrigation agriculture and urban landscaping in various bioclimatic conditions.

Experiments with grass lawns in arid climates (U.A.E., Dubai) and the Moscow metropolis have shown an increase in the storage of productive water and grass biomass by 2 times or more under the influence of gel-forming soil conditioners. The capillary gel barrier in the topsoil, along with a crushed stone screen between the topsoil and subsoil, reliably protects the rhizosphere from secondary salinization, blocking the upward salt flows to the evaporating soil surface.

Experiments with potato crops in arid conditions of Uzbekistan and in the temperate climate of the Moscow region using flood and drip irrigation revealed an increase in the yield of tubers from 30% and above with 30–50% savings in irrigation water under the influence of gel-forming soil conditioners and their compositions with biocides (ions, silver nanoparticles, organic fungicide). Regardless of the method of using gel biocides (introduction into an intermicellar solution for hydrogel swelling or embedding directly into a polymer matrix during synthesis), their application in the rhizosphere reliably protects the soil and tubers from the main pathogens, including late blight, capable of causing great damage (25–30% and more) to the potato crop.

All these positive effects require sufficiently large amounts of gel-forming soil conditioners, ranging from 100–300 kg/ha for potato growing to 1–2 t/ha for continuous lawn coverings in urban landscaping. In this regard, future challenges in our opinion should include lowering the cost of gel-forming soil conditioners and combining the most important technological properties into one material (water retention, soil aggregation and erosion protection, fixing agrochemicals, trace elements, and biocides), as well as increasing the sustainability of these materials to adverse environmental factors, primarily biodegradation.

## 6. Patents

The results of the work are used in the synthesis technology of biodegradation-resistant filled hydrogels patented in the Russian Federation: 

Patent RU №2726561 (https://findpatent.ru/patent/272/2726561.html, accessed on 14 October 2022).

Patent RU 2639789 (http://www.findpatent.ru/patent/263/2639789.html, accessed on 14 October 2022).

## Figures and Tables

**Table 1:** Granulometric composition of upper (0–20 cm) soil horizons.

Granulometric Fractions:	Soils:		
	Carbonate Loamy–Sandy Arenosol from the Emirate of Dubai, U.A.E.	Loamy–Sandy Retisol from the Moscow Region, Russia	Loamy Serozem from the Tashkent Region, Uzbekistan
Clay (<2 µm)	4.2	2.4	20.7
Silt (2–50 µm)	23.3	24.6	45.6
Very fine sand (50–100 µm)	22.4	15.4	13.3
Fine sand (100–250 µm)	39.2	36.0	8.9
Medium sand (250–500 µm)	9.6	17.0	6.4
Coarse sand (500–1000 µm)	1.3	4.6	5.1
Very coarse sand (1000–2000 µm)	0	0	0

## References

[1] Sojka R.E., Bjorneberg D.L., Entry J.A. (2007). Polyacrylamide in Agriculture and Environmental Land Management. Adv. Agron..

[2] Behera S., Mahanwar P.A. (2020). Superabsorbent Polymers in Agriculture and Other Applications: A review. Polym. Plast. Technol. Mat..

[3] Wu L., Liu M., Rui-Liang R.L. (2008). Preparation and Properties of a Double-coated slow-release NPK Compound Fertilizer with Superabsorbent and Water-retention. Biores. Technol..

[4] Smagin A., Sadovnikova N., Smagina M. (2019). Synthetic Gel Structures in Soils for Sustainable Potato Farming. Sci. Rep..

[5] Campos E.V.R., de Oliveira J.L., Fraceto L.F., Singh B. (2015). Polysaccharides as Safer Release Systems for Agrochemicals. Agron. Sustain. Dev..

[6] Lentz R.D., Andrawes F.F., Barvenik F.W., Koehn A.C. (2008). Acrylamide Monomer Leaching from Polyacrylamide-treated Irrigation Furrows. J. Environ. Qual..

[7] Novoskoltseva O.A., Panova I.G., Loiko N.G., Nikolaev Y.A., Litmanovich E.A., Yaroslavov A.A. (2021). Polyelectrolytes and Polycomplexes for Stabilizing Sandy Grounds. Polym. Sci. Ser. B.

[8] Smagin A.V., Budnikov V.I., Sadovnikova N.B., Kirichenko A.V., Belyaeva E.A., Krivtsova V.N. (2022). Gel-Forming Soil Conditioners of Combined Action: Laboratory Tests for Functionality and Stability. Polymers.

[9] Al-Darby A.M. (1996). The Hydraulic Properties of a Sandy Soil Treated with Gel-forming Soil Conditioner. Soil Technol..

[10] El-Rehim H.A.A., Hegazy E.S.A., El-Mohdy H.L.A. (2004). Radiation Synthesis of Hydrogels to Enhance Sandy Soils Water Retention and Increase Plant Performance. J. Appl. Polym. Sci..

[11] Al-Darby A.M., Al-Asfoor S.I., El-Shafei Y.Z. (2002). Effect of Soil Gel-Conditioner on the Hydrophysical Properties of Sandy Soil. J. Saudi Soc. Agric.Sci..

[12] Shahid S.A., Qidwai A.A., Anwar F., Ullah I., Rashid U. (2012). Improvement in the Water Retention Characteristics of Sandy Loam Soil Using a Newly Synthesized Poly(acrylamide-co-acrylic Acid)/AlZnFe_2_O_4_ Superabsorbent Hydrogel Nanocomposite Material. Molecules.

[13] Yang L., Yang Y., Chen Z. (2014). Influence of Super Absorbent Polymer on Soil Water Retention, Seed Germination and Plant Survivals for Rocky Slopes Eco-engineering. Ecol. Eng..

[14] Banedjschafie S., Durner W. (2015). Water Retention Properties of a Sandy Soil with Superabsorbent Polymers as Affected by Aging and Water Quality. J. Plant Nutr. Soil Sci..

[15] Campos E.V.R., de Oliveira J.L., Fraceto L.F. (2014). Applications of Controlled Release Systems for Fungicides, Herbicides, Acaricides, Nutrients, and Plant Growth Hormones: A Review. Adv. Sci. Eng. Med..

[16] Ghani A.A., Shahzad A., Moztahida M., Tahir K., Jeon H., Kim B., Lee D.S. (2020). Adsorption and Electrochemical Regeneration of Intercalated Ti_3_C_2_Tx MXene for the Removal of Ciprofloxacin from Wastewater. Chem. Eng. J..

[17] Xu X., Bizmark N., Christie K.S.S., Datta S.S., Ren Z.J., Priestley R.D. (2022). Thermoresponsive Polymers for Water Treatment and Collection. Macromolecules.

[18] Guo Y., Guan W., Lei C., Lu H., Shi W., Yu G. (2022). Scalable super hygroscopic polymer films for sustainable moisture harvesting in arid environments. Nat. Com..

[19] Mikula P., Mlnaříková M., Nadres E.T., Takahashi H., Babica P., Kuroda K., Bláha L., Sovadinová I. (2021). Synthetic Biomimetic Polymethacrylates: Promising Platform for the Design of Anti-Cyanobacterial and Anti-Algal Agents. Polymers.

[20] Smagin A.V., Sadovnikova N.B., Vasenev V.I., Smagina M.V. (2018). Biodegradation of Some Organic Materials in Soils and Soil Constructions: Experiments, Modeling and Prevention. Materials.

[21] Johnson M.S. (1984). Effect of Soluble Salts on Water Absorption by Gel-forming Soil Conditioners. J. Sci. Food Agric..

[22] Kazanskii K.S., Dubrovskii S.A. (1992). Chemistry and Physics of Agricultural Hydrogels. Adv. Polym. Sci..

[23] Vorobieva E.V. (2020). Swelling of Polyacrylamide-based Hydrogel in Aqueous Solutions of Low-molecular Salts. Dokl. Nat. Acad. Sci. Belarus.

[24] Hadas A., Kautsky L., Goek M., Kara E.E. (2004). Rates of Decomposition of Plant Residues and Available Nitrogen in Soil, Related to Residue Composition Through Simulation of Carbon and Nitrogen Turnover. Soil Biol. Biochem..

[25] Lande S.S., Bosch S.J., Howard P.H. (1979). Degradation and Leaching of Acrylamide in soil. J. Environ. Qual..

[26] Abdelmagid H.M., Tabatabai M.A. (1982). Decomposition of Acrylamide in Soils. J. Environ. Qual..

[27] Shanker R., Ramakrishna C., Seth P.K. (1990). Microbial Degradation of Acrylamide Monomer. Arch. Microb..

[28] Kay-Shoemake J.L., Watwood M.E., Sojka R.E., Lentz R.D. (1998). Polyacrylamide as a Substrate for Microbial Amidase in Culture and in Soil. Soil Biol. Biochem..

[29] Sojka R.E., Entry J.A. (2000). Influence of Polyacrylamide Application to Soil on Movement of Microorganisms in Runoff Water. Environ. Pollut..

[30] Oladosu Y., Rafii M.Y., Arolu F., Chukwu S.C., Salisu M.A., Fagbohun I.K., Muftaudeen T.K., Swaray S., Haliru B.S. (2022). Superabsorbent Polymer Hydrogels for Sustainable Agriculture: A Review. Horticulturae.

[31] Akhter J., Mahmood K., Malik K.A., Mardan A., Ahmad M., Iqbal M.M. (2004). Effects of Hydrogel Amendment on Water Storage of Sandy Loam and Loam Soils and Seedling Growth of Barley, Wheat, and Chickpea. Plant Soil Environ..

[32] Koupai A.J., Asadkazemi J. (2006). Effects of a Hydrophilic Polymer on the Field Performance of an Ornamental Plant (*Cupressus arizonica*) under Reduced Irrigation Regimes. Iran. Polym. J..

[33] Yangyuoru M., Boateng E., Adiku S.G.K., Acquah D., Adjadeh T.A., Mawunya F. (2006). Effects of Natural and Synthetic Soil Conditioners on Soil Moisture Retention and Maize Yield. J. App. Eco..

[34] Yazdani F., Allahdadi I., Akbari G.A. (2007). Impact of Superabsorbent Polymer on Yield and Growth Analysis of Soybean (*Glycine max* L.) under Drought Stress Condition. Pak. J. Biol. Sci..

[35] Rahman A., Ahmad R., Safdar M. (2011). Effect of Hydrogel on the Performance of Aerobic Rice Sown under Different Techniques. Plant Soil Environ..

[36] Moghadam T., Shirani-Rad H.R., Mohammadi A.H.N., Habibi G., Modarres S., Mashhadi S.A.M., Dolatabadian M.A. (2013). Response of Six Oilseed Rape Genotypes to Water Stress and Hydrogel Application. Pesqui. Agropecuária Trop..

[37] Scremin O.B., da Silva A.G., de Mamann A.T.V., Mantai R.D., Brezolin A.P. (2017). Nitrogen Efficiency in Oat Yield through the Biopolymer Hydrogel. Rev. Bras. Eng. Agríc. Ambient..

[38] Dar S.B., Mishra D., Zahida R., Afshana B.B. (2017). Hydrogel: To Enhance Crop Productivity Per Unit Available Water under Moisture Stress Agriculture. Bull. Environ. Pharma. Life Sci..

[39] Rezashateri M., Khajeddin S.J., Matinkhah S.H., Majidi M.M. (2017). The Effects of Soil Ameliorating Hydrogels on Root System Characteristics of *Avena fatua* in Two Different Soil Textures. J. Water Soil Sci..

[40] Abd El-Aziz G.H., Ibrahim A.S., Fahmy A.H. (2022). Using Environmentally Friendly Hydrogels to Alleviate the Negative Impact of Drought on Plant. Open J. Appl. Sci..

[41] Rajanna G.A., Manna S., Singh A., Babu S., Singh V.K., Dass A., Chakraborty D., Patanjali N., Chopra I., Banerjee T. (2022). Biopolymeric Superabsorbent Hydrogels Enhance Crop and Water Productivity of Soybean–Wheat System in Indo-Gangetic Plains of India. Sci. Rep..

[42] Smagin A.V., Sadovnikova N.B. (2015). Creation of Soil-Like Constructions. Eur. Soil Sci..

[43] Lentz R.D., Sojka R.E. (2009). Long-term Polyacrylamide Formulation Effects on Soil Erosion, Water Infiltration, and Yields of Furrow-Irrigated Crops. Agron. J..

[44] Vasenev V.I., Smagin A.V., Ananyeva N.D., Ivashchenko K.V., Gavrilenko E.G., Prokofeva T.V., Patlseva A., Stoorvogel J.J., Gosse D.D., Valentini R., Rakshit A., Abhilash P.C., Singh H.B., Ghosh S. (2017). Urban Soil’s Functions: Monitoring, Assessment, and Management. Adaptive Soil Management: From Theory to Practices.

[45] Rizwan M., Gilani S.R., Durani A.I., Naseem S. (2021). Materials diversity of hydrogel: Synthesis, polymerization process and soil conditioning properties in agricultural field. J. Adv. Res..

[46] Fedotov G.N., Dobrovolskii G.V. (2012). Soil Gels and Their Research. Dokl. Biol. Sci..

[47] Krutyakov Y.A., Kudrinskiy A.A., Zherebin P.M., Yapryntsev A.D., Pobedinskaya M.A., Ebansky S.N., Denisov A.N., Mikhaylov D.M., Lisichkin G.V. (2016). Tallow Amphopolycarboxyglycinate-Stabilized Silver Nanoparticles: New Frontiers in Development of Plant Protection Products with a Broad Spectrum of Action Against Phytopathogens. Mater. Res. Express.

[48] Richards L.A. (1954). Diagnosis and Improvement of Saline and Alkali Soils.

[49] Smagin A.V., Sadovnikova N.B., Belyaeva E.A., Kirichenko A.V., Krivtsova V.N. (2021). Capillary Effects in Polydisperse Systems and Their Use in Soil Engineering. Eur. Soil Sci..

[50] Smagin A.V. (2021). Thermodynamic Concept of Water Retention and Physical Quality of the Soil. Agronomy.

[51] Asimovic Z., Cengic L., Hodzic J., Murtic S. (2016). Spectrophotometric Determination of Total Chlorophyll Content in Fresh Vegetables. Godina LXI Broj.

[52] GenBit LLC. http://genbitgroup.com/.

[53] VNIIF. http://http://vniif.ru/.

[54] Kingsland S. (1982). The Refractory Model: The Logistic Curve and the History of Population Ecology. Q. Rev. Biol..

[55] PC-Progress. https://www.pc-progress.com/.

[56] Lagutina M.A., Dubrovskii S.A. (1996). The Swelling Pressure of Weakly Ionic Acrylamide Gels. Polym. Sci. Ser. A.

[57] Bakr D.I., Al-Khalidi J., Hadi A.S. (2021). Comparison of Some Mathematical Models to Calculate Evapotranspiration in Contrasting Regions of Iraq. Environ. Asia.

[58] Darrah P.R. (1991). Models of the rhizosphere: I. Microbial Population Dynamics Around a Root Releasing Soluble and Insoluble Carbon. Plant Soil.

[59] Khamiraev U.K. (2018). Availability of *Phytophthora infestans* (*Mont*.) *de Bary* on the Uzbekistan territory and modern fungicides application to control it. Bull. Sci. Pract..

[60] Chevillard A., Angellier-Coussy H., Guillard V., Gontard N., Gastaldi E. (2012). Controlling Pesticide Release via Structuring Agropolymer and Nanoclays Based Materials. J. Hazard. Mater..

[61] Dhanapal V., Subramanian K. (2021). Superabsorbent polymers: A state-of-art review on their classification, synthesis, physicochemical properties, and applications. Rev.Chem. Eng..

[62] Kudryavskii D.L., Fomina E.K., Krul L.P., Yakimenko O.V. (2020). Swelling of a hydrogel based on a copolymer of acrylamide and sodium acrylate in aqueous solutions copper (II) chloride with amino acid additives. Weight. Natl. Acad. Sci. Belarus. Ser. Chem..

[63] Shirinov S.D., Jalilov A.T. (2018). Investigation of the swelling kinetics of synthesized hydrogels based on hydrolyzed polyacrylonitrile. Univers. Chem. Biol. Electron. Sci. J..

[64] Louf J.-F., Lu N.B., O’Connell M.G., Cho H.G., Datta S.S. (2021). Under pressure: Hydrogel swelling in a granular medium. Sci. Adv..

[65] Misiewicz J., Głogowski A., Lejcuś K., Marczak D. (2020). The Characteristics of Swelling Pressure for Superabsorbent Polymer and Soil Mixtures. Materials.

